# Supporting social prescribing in primary care by linking people to local assets: a realist review

**DOI:** 10.1186/s12916-020-1510-7

**Published:** 2020-03-13

**Authors:** Stephanie Tierney, Geoff Wong, Nia Roberts, Anne-Marie Boylan, Sophie Park, Ruth Abrams, Joanne Reeve, Veronika Williams, Kamal R. Mahtani

**Affiliations:** 1 Nuffield Department of Primary Care Health Sciences, University of Oxford, Oxford, UK; 2 Bodleian Health Care Libraries, University of Oxford, Oxford, UK; 3 Department of Primary Care and Population Health, University College London, London, UK; 4 Hull York Medical School, University of Hull, Hull, UK; 5 School of Nursing, Nipissing University, North Bay, Canada

**Keywords:** Realist review, Social prescribing, Link workers, Care navigators, Social capital, Evidence synthesis

## Abstract

**Background:**

Social prescribing is a way of addressing the ‘non-medical’ needs (e.g. loneliness, debt, housing problems) that can affect people’s health and well-being. Connector schemes (e.g. delivered by care navigators or link workers) have become a key component to social prescribing’s delivery. Those in this role support patients by either (a) signposting them to relevant local assets (e.g. groups, organisations, charities, activities, events) or (b) taking time to assist them in identifying and prioritising their ‘non-medical’ needs and connecting them to relevant local assets. To understand how such connector schemes work, for whom, why and in what circumstances, we conducted a realist review.

**Method:**

A search of electronic databases was supplemented with Google alerts and reference checking to locate grey literature. In addition, we sent a Freedom of Information request to all Clinical Commissioning Groups in England to identify any further evaluations of social prescribing connector schemes. Included studies were from the UK and focused on connector schemes for adult patients (18+ years) related to primary care.

**Results:**

Our searches resulted in 118 included documents, from which data were extracted to produce context-mechanism-outcome configurations (CMOCs). These CMOCs underpinned our emerging programme theory that centred on the essential role of ‘buy-in’ and connections. This was refined further by turning to existing theories on (a) social capital and (b) patient activation.

**Conclusion:**

Our realist review highlights how connector roles, especially link workers, represent a vehicle for accruing social capital (e.g. trust, sense of belonging, practical support). We propose that this then gives patients the confidence, motivation, connections, knowledge and skills to manage their own well-being, thereby reducing their reliance on GPs. We also emphasise within the programme theory situations that could result in unintended consequences (e.g. increased demand on GPs).

## Background

There is an urgent need to rethink how general practice can manage rising workload demands without compromising quality and patient safety [1]. Up to one in five cases seen by a general practitioner (GP) are for difficulties that could be classed as ‘non-medical’ (e.g. inadequate housing, financial issues, bereavement, loneliness) [2]. In recognition of these wider determinants of well-being, social prescribing has become a core component of current and future National Health Service (NHS) policy and practice to deliver person-centred care [3] and reduce GP workload [4].

GPs have historically signposted patients to organisations and groups to support their ‘non-medical’ needs. However, workload pressures and changing landscape of the voluntary and community sector (VCS) make this difficult to sustain [5, 6]. Consequently, the United Kingdom (UK) has been at the forefront of formalising the use of social prescribing alongside traditional medical treatment within primary care, to address the environmental, economic, social and psychological issues affecting people’s well-being. Similar initiatives, often building on work within the UK, have been developed across other high-income countries, including Canada, America, Australia and New Zealand.

To facilitate and sustain delivery of social prescribing, services we define in this paper as ‘connector schemes’ have been introduced, whereby individuals help patients to access support to meet their ‘non-medical’ needs by linking them to local assets (e.g. groups, organisations, charities, activities, events). Various incarnations have emerged across the UK, differing in levels of interaction with a patient [7]. For example, active signposting involves existing members of staff at a surgery (generally receptionists) giving information to patients about local sources of help. Active signposting has been described as ‘light touch’ social prescribing that ‘works best for people who are confident and skilled enough to find their own way to services after a brief intervention’ [8]. Staff delivering this form of connector scheme have been described as ‘care navigators’ [9]. Other services allow for more prolonged contact with a patient, who is helped to produce an action plan and ways of meeting personal goals within it by drawing on local assets. These more intensive approaches usually involve people employed specifically to undertake this work. In England, the term ‘link worker’ tends to be used to refer to this type of connector scheme in policy documents. For example, link workers are mentioned in the NHS long-term plan, which makes a commitment to funding this role within primary care networks (PCNs) (these are amalgamations of several GP practices, serving a population of 30,000–50,000) [10].

Social prescribing connector schemes can be regarded as a complex intervention because they consist of a range of components (e.g. educating, encouraging, empowering people), include several stakeholders (e.g. patients, VCS, primary care staff, link workers) and have variable outcomes (e.g. at the patient level, the surgery level, the health service level). Guidance from the Medical Research Council [11] recognises that successful implementation and maintenance of a complex intervention requires a clear conceptual/theoretical model outlining the components and how they work together to produce intended outcomes. Previous reviews focused on social prescribing and connector schemes [12–14] have not sought to understand mechanisms producing outcomes and how context (macro or micro) might be shaped by the introduction of a link worker or care navigator. A previous realist review by Husk et al. [15] proposed a model for understanding social prescribing more generally, generating statements about enrolment, engagement and completion of activities. Our review has a more specific focus on the link worker role, because these individuals have been identified as key to social prescribing delivery and are being implemented across primary care in England. It sought to answer the following questions:

- What are the outcomes associated with social prescribing connector schemes in primary care?
- What are the mechanisms that produce these outcomes?
- Under what conditions (context) are these mechanisms activated?

Realist reviews are a theory-driven approach to synthesising existing data. They are underpinned by a realist philosophy of science and develop causal explanations of outcomes. Such explanations focus on mechanisms, and contexts required to trigger them—resulting in the development, refinement and testing of context-mechanism-outcome configurations (CMOCs) (see Table 1 for definitions). CMOCs are embedded within a programme theory (a proposition about how an intervention is thought to work, under what conditions) [16]. Our review offers an understanding of how social prescribing connector schemes might work in practice, for whom, in what circumstances and how to optimise their delivery within primary care. Reviewing the evidence on this type of service is warranted given the prominence and recent investment in link workers within the NHS and the introduction of social prescribing in other countries.

**Table 1:** Definitions of context, mechanism and outcome

***Context:*** Influences whether a mechanism is triggered or not. It may include macroeconomic conditions, cultural practices and interpersonal relations. Pawson [16] suggests that understanding context can include a focus on ‘(i) the individual players, (ii) their interrelationships, (iii) the institutional location and (iv) the surrounding infrastructure.’	
***Mechanism:*** Refers to what it is about social prescribing connector roles that cause outcomes. Mechanisms tend to be unobservable, ‘embodied in the subjects’ reasoning…’ [16]. Most usually in health services research, they can be conceptualised as a response (e.g. fear, reputation management, feeling valued, needing to appear competent) to resources provided by an intervention.	
***Outcome:*** From a realist perspective, ‘variations in programme performance are a crucial first step but outcome patterns considered alone are only surface “markers” or “traces”… the potential outward signals of inner workings of a programme in a particular manifestation’ [16]. Hence, we were not so much interested in the percentage or degree to which link workers/care navigators had worked (or not) but in how different outcomes were produced under different contexts.	
***Context-mechanism-outcome configuration (CMOC):*** This is the way in which causal explanations are presented in realist reviews. They are propositions that explain how an outcome is caused (O) ‘because of the action of some underlying mechanisms (M), which only comes into operation in particular contexts (C)’ [16]. CMOCs should be phrased in a manner that is testable.	

## Methods

Our realist review ran from April 2018 to June 2019; the protocol was registered with PROSPERO (CRD42018095090). Throughout, stakeholders provided us with feedback and advice on our initial ideas and later emerging analyses. Their feedback helped us comprehend and establish the relevance of our findings. At the start, we discussed social prescribing connector schemes with those delivering or referring to such a service (*n* = 12). In addition, we spoke to eight members of the public (our PPI group) with whom we continued to meet as the review progressed. We also talked about the review’s findings to other stakeholders (*n* = 41) at arranged meetings and conferences.

We were guided by the five steps for conducting a realist review outlined by Pawson et al. [17] and reported following the RAMESES publication standards [18].

### Step 1: Clarifying scope

**Fig. 1:**
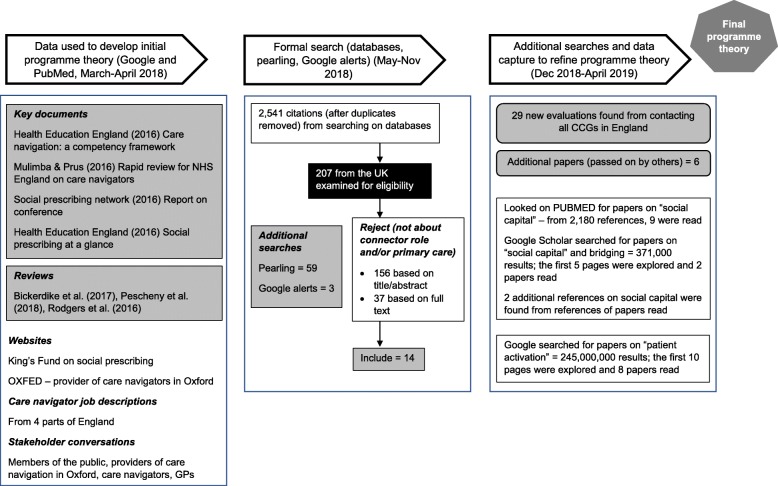
Processes and literature searches used to develop CMOCs and the emerging and final programme theory (grey boxes in the diagram highlight references that were coded in step 3 of the review)

This step entails deciding on the research questions, being clear about the purpose of the review and developing an initial programme theory (see Additional file 1: Figure of initial programme theory). To develop the initial programme theory, we read policy and other documents, looked on key websites, examined job descriptions, watched videos produced by NHS England and talked to stakeholders (see Fig. 1). When we planned the review, the term ‘care navigator’ was current for a connector role and covered a range of levels of interaction with patients [19]. However, during the review’s production, terminology evolved. As mentioned above, in recent NHS policy documents, ‘link worker’ is used to depict those who have a more sustained interaction with patients and ‘care navigator’ for a less intensive signposting role. Many of the CMOCs we developed for the review relate specifically to the more involved model delivered by link workers. Therefore, we tend to use the term ‘link worker’ within this paper. Nevertheless, some of the broad concepts detailed below cut across various types of social prescribing connector schemes (i.e. care navigators as well as link workers).

### Step 2: Search for evidence

#### Formal search

The initial programme theory functioned as a springboard for our formal search for data. An information specialist piloted a search strategy; after feedback and amendments, she ran a full search on the following databases: ASSIA, CINAHL, Cochrane Database of Systematic Reviews, Cochrane Central Register of Controlled Trials, DARE, Dissertations and Abstracts, EMBASE, HMIC, MEDLINE, SCI and SSCI. We searched using terms related to link workers/care navigators and general practice; free-text and indexing terms were included (see Additional file 2: Search conducted on MEDLINE). Databases were searched for literature published until the end of May 2018.

This formal search resulted in 2541 references; most were from America, where care navigation has been linked closely to oncology, clinical co-ordination and screening attendance. These were excluded because they did not meet the review’s inclusion criteria (see Table 2). We used ‘pearling’ (where we examined the reference list of finally included relevant articles) to identify additional documents. We were also sent 3 articles by colleagues who knew we were doing the review. We set up Google alerts to find papers using the following terms: (navigator* OR navigation OR “link worker*”) AND (“primary care” OR “general practice” OR GP*). These alerts were stopped at the end of November 2018, when saturation of concepts had started to be established.

**Table 2:** Inclusion and exclusion criteria

Inclusion	Exclusion
Written in English	Non-UK focused
About social prescribing connector roles	Focused on clinical navigation
Relate to primary care	
Focuses on adults (18+ years)	

#### Additional searches

At the end of 2018, we sought to identify evaluations of care navigator/link worker schemes from Clinical Commissioning Groups (CCGs) in England. We contacted all 195 CCGs [7] and, using a Freedom of Information request, invited them to forward any evaluations of services in their area. We had not originally planned this piece of work, but since many references we found were evaluations (rather than published studies), we thought a systematic approach to locating such documents was appropriate. This resulted in 29 extra evaluations being included in the review. Evaluations located from CCGs contributed the following unique elements to our programme theory:

- Having someone with passion and drive to spearhead a social prescribing connector scheme can persuade others to accept it, but this person must be seen as credible to be heard and trusted = fits with the broader theme of ‘buy-in’ (see below)
- Patients feeling cared for by the link worker = fits with the broader theme of connection (see below)
- Not having long waiting lists to see a link worker because this can fracture trust = fits with the ideas of ‘buy-in’ and connection (see below).

#### Screening

Realist reviews are inclusive in terms of document type. As a broad principle, quantitative data show patterns that inform thinking about what needs explaining in a programme theory, whilst qualitative studies are more likely to contain data that explain patterns and how outcomes may occur—thus contributing to different aspects of programme theory development. Realist reviews can include grey literature (e.g. commentaries, editorials, evaluations or blogs), which may hold information for constructing CMOCs.

Our inclusion and exclusion criteria for consideration in step 3 are shown in Table 2. We centred on social prescribing connector roles in the UK, as we wished to explore how this intervention might work in the NHS. All references were stored on Endnote, where decisions about inclusion and exclusion were recorded. Screening of titles and abstracts, to see if they met the inclusion criteria, was undertaken by ST. A random sample of 10% of citations was also reviewed by KM or GW to establish there had been consistency in the application of these criteria. Three abstracts resulted in disagreement, due to the differences in the scope of what constitutes a social prescribing connector role; this was resolved through discussion.

### Step 3: Appraising papers and extracting data

Papers meeting the inclusion criteria, or if this was unclear from the title and abstract, were read in full to judge whether they were ‘fit for purpose’—containing useful data for developing or testing our emerging CMOCs/programme theory (relevance) and examining, when necessary, whether the piece of data used was underpinned by credible and trustworthy methods (rigour). This meant that when a CMOC was based on a limited amount of data, we examined in more detail the methods used. We made judgments about the ‘rigour’ of our final programme theory by assessing its explanatory powers, using the criteria of consilience (accounting for as much of the data as possible), simplicity (not containing lots of caveats) and analogy (relating to what is already known) [20].

Data extraction was undertaken by ST; 10% of the coded documents were reviewed independently by KM or VW. As well as checking for systematic errors, this process and discussion between researchers helped to bring in alternative ideas. Characteristics of included documents were recorded in an Excel file. Full-text documents were stored and coded in NVIVO. Coding was largely inductive, although consideration of concepts within the initial programme theory allowed for a degree of deductive coding, as did discussions with key stakeholders. Early coding was based on descriptive concepts (e.g. recruitment and retention of link workers, understanding of the role, communication across professional boundaries, adequate community resources). These were then explored by ST to identify elements that could help with building CMOCs (see step 4 below and Additional file 3: CMOCs for programme theory refinement, with data extracts).

### Step 4: Synthesising data

Our analytic process started by considering an outcome (e.g. patient having a more positive outlook). We then used our interpretations of data to develop explanations of how different mechanisms might have been triggered in a specific context to cause the outcome; the context in a realist review can incorporate pre-existing, macroeconomic conditions; institutional norms; or interpersonal relations [21]. Potential CMOCs were created by ST. These were shared and discussed with KM and GW. We considered CMOCs alongside our emerging programme theory, refinement of which continued as we progressed. Memos were made to record evolving understanding. To illustrate and guide our findings, we created diagrams that were drafted and reworked to include several iterations of CMOCs. We continued this stage of the analysis until CMOCs reflected the wide range of data contained within documents coded for the review.

#### Engagement with substantive theory

Whilst extracting data from included documents, we logged whether they referenced substantive theories. After inductive coding of papers located from the formal search (see Fig. 1) had been completed, and as our programme theory became more nuanced, we focused on theories we felt were pertinent in explaining key elements of it. We identified 2 substantive theories that were referenced in 33 of our coded documents. They acted as lenses through which to deepen our understanding of social prescribing connector roles. We carried out exploratory searches for key documents to augment our knowledge of these theories and how they might relate to link workers (see Fig. 1 for details).

### Step 5: Disseminating and implementing evidence

Alongside writing up results for publication and describing them in presentations, this stage involved sharing our CMOCs and programme theory with key stakeholders.

## Results

**Fig. 2:**
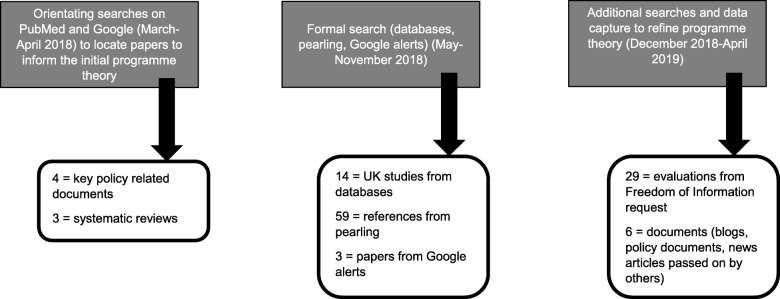
How the 118 documents coded in step 3 to help with developing CMOCs were located

**Fig. 3:**
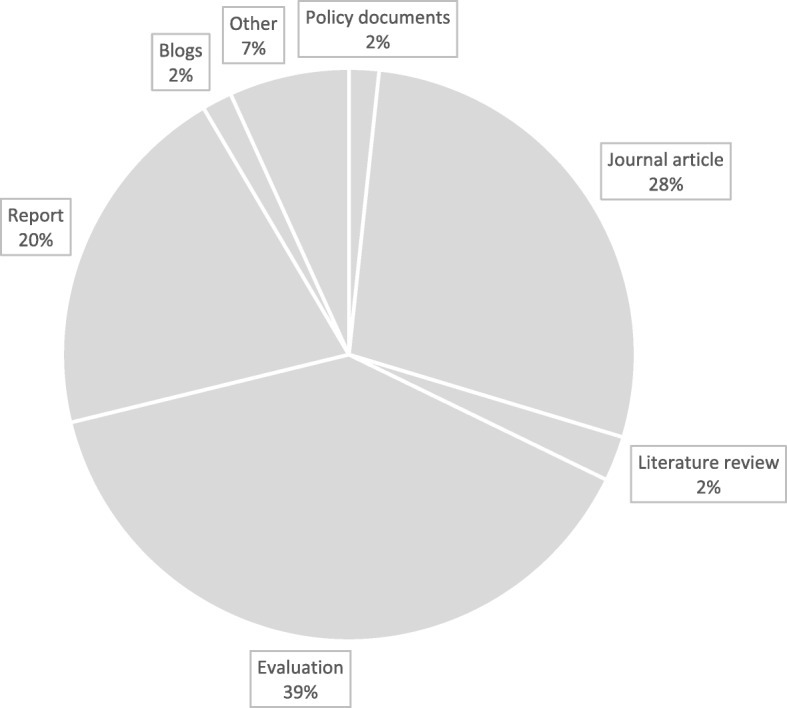
Type of documents coded to develop CMOCs

In total, 118 documents (see Fig. 2 for details on how they were located and Additional file 4: Table of studies coded in the review) were coded to develop CMOCs that were used to refine and expand our initial programme theory. They were published between 1992 and 2019, covering projects run in the south, midlands and north of England, as well as Scotland, Wales and Northern Ireland. As illustrated in Fig. 3, most documents used to develop CMOCs could be classed as ‘grey literature’, coming from evaluations and reports.

In this section, we provide a narrative overview of the two key concepts underpinning our final programme theory. It is based on the initial 29 CMOCs we developed (see Additional file 3: CMOCs for programme theory refinement, with data extracts) from the reviewed literature.

### Concept 1: Creating and sustaining ‘buy-in’

Social prescribing connector roles are relatively new to the NHS. ‘Buy-in’ to this type of service and people delivering it is the important first step to producing intended outcomes. This relates to legitimising the service and a belief in individuals undertaking this role.

#### Legitimising the service

Key stakeholders (e.g. patients, GPs, commissioners, primary care staff) must ‘buy-in’ to the social prescribing connector role as a judicious route to addressing ‘non-medical’ needs; otherwise, the service may be dismissed by patients as a means of blocking them from seeing their GP, and by healthcare professionals (HCPs) as another ‘gimmick’ (a danger if funding is short-term and the service is branded as a pilot). Acceptance may be engendered through the endorsement of such provision by credible sources. For example, HCPs may hear about a service in glowing terms from respected colleagues or those in a leadership role. ‘Buy-in’ may be exhibited in a practice setting by senior staff making space for a link worker (e.g. providing them with a room to see patients, giving them access to tea/coffee making facilities).

Patients may view link workers favourably after discussing the role with a trusted GP, whose referral validates the service. However, it may be necessary for patients to be at a stage in life when they have the energy and mental capacity to ‘buy-in’ to a new way of addressing their problems. This may not be possible if psychosocial difficulties (e.g. family complications or poor mental health) are perceived as overwhelming. Not having to wait to see a link worker is also crucial, to prevent a patient losing momentum in seeking assistance.

Clear information about the role and remit of link workers should avoid misunderstandings and unrealistic expectations. Patients may be deterred from seeing a link worker if they believe this is part of formal social services, regard referral as stigmatising or feel that their situation requires medical intervention. Making time to explain the service to HCPs and patients (e.g. in face-to-face meetings, via accessible leaflets) is therefore important. Likewise, incorporating processes (e.g. referrals) into existing systems within a surgery (e.g. linking it to current IT platforms) will encourage HCPs to see the service as easy to use. Consulting with key stakeholders from the outset could increase the chance of social prescribing connector roles being developed in a manner that helps rather than hinders current practice.

Members of the VCS must ‘buy-in’ to the idea of a social prescribing connector role, believing they will receive appropriate referrals and that demand for their services will remain manageable. The VCS should be involved in initial discussions about setting up a connector scheme, to forestall concerns and to ensure those working in this arena feel like valued partners—thereby helping to foster ‘buy-in’.

#### Belief in an individual link worker

Key stakeholders must ‘buy-in’ to the skills and knowledge of individual link workers. HCPs need to regard them as credible and competent before forwarding referrals. This can be achieved through receiving regular feedback about how a link worker is helping patients. Positive feedback creates confidence in the link worker. A feedback loop may then be established, increasing HCPs’ trust in this individual, so they make more referrals.

Patients must believe they will benefit from seeing a link worker. For this to occur requires link workers with the skills, attitude and time to encourage patients to open up, who demonstrate a genuine wish to help by offering personalised support. They may start by working on simpler difficulties to resolve (e.g. arranging for mobility equipment to be installed into someone’s home), before tackling more challenging issues (e.g. social isolation following bereavement), so that patients lacking motivation to change are not discouraged and to enable people to experience incremental successes. Link workers may need prolonged engagement with a patient to work in this way. The service is at risk of dilution if the workload is too great; this could prevent link workers from thinking creatively about how best to support individuals and from establishing connections within the VCS, which is important for ‘buy-in’ to an individual link worker’s credibility.

### Concept 2: Establishing and maintaining connections

‘Buy-in’ is a first, essential step in establishing a social prescribing connector scheme and ensuring that patients are willing to try it. A further issue is ongoing inter-relations. This starts when trying to secure the ‘buy-in’ of stakeholders but warrants further consideration, as once the service has been accepted as a viable option, its success rests on sustained, strong connections between the link worker and other key stakeholders.

#### Giving life meaning and inspiring hope

The ‘buy-in’ referred to above results in a patient who is prepared to listen to what a link worker proposes. Link workers give people permission to consider and prioritise their needs and legitimises the accessing of support from others. They can help lessen the mental load associated with change by developing an action plan with the patient. Feeling safe to disclose potentially sensitive information to a link worker may be a stage that patients have to pass through; ongoing conversations allow trust to be iteratively built and reinforced, prompting patients to try new activities or to seek external help. Patients may consider, with the link worker, ways of resolving potential barriers (e.g. due to travel, childcare). This enables them to move forward in life, becoming connected to community resources, so they feel less isolated and more in control of their situation. Making new connections through the link worker can result in patients no longer fixating on personal problems. There is a danger of patients becoming dependent on a link worker as *the* source of support; this should be tempered if individuals create new and meaningful connections within the community, which may include reconnecting with friends and family because of a more positive outlook on life. Such an improved outlook may encourage those with existing health conditions to actively engage in self-care.

#### Integrating health and community assets

Link workers were depicted in some reviewed literature as fostering better connections between HCPs and the VCS. It was noted that the former can be sceptical and unsure about the latter’s ability to help patients. In addition, VCS staff can feel that access to communicate with HCPs is challenging. Link workers undertake a brokerage role, having time to foster relationships within each setting and understanding the culture and language associated with primary care and the VCS. They can forge links by organising joint events, or they may produce feedback about patients’ progress that is shared with both groups. This raises awareness of the work and input of each party in addressing patients’ needs, increasing mutual respect. HCPs can feel less frustrated when managing patients with ‘non-medical’ problems, if a link worker highlights options available in the VCS.

#### Supporting the supporter

For link workers to continue acting as a credible source of assistance for patients, they should receive appropriate training (e.g. in active listening, being non-judgmental, motivational techniques). An environment offering supervision or peer support allows anxieties or difficulties associated with the role to be shared and explored. Problems arise when the link worker’s capacity and capabilities are overextended, especially if HCPs refer complex cases because (a) they believe the link worker can cope and (b) there is a lack of immediately accessible alternatives (due to long waiting lists for statutory services). The link worker may become so overstretched that they leave their post. When a link worker leaves, they take with them tacit knowledge of local, reliable VCS providers, and relational links. Consequently, improvements made by the service may temporarily decline as a new link worker is installed and has to create positive connections with a range of stakeholders.

#### Intermediate programme theory (prior to considering existing, substantive theories)

**Fig. 4:**
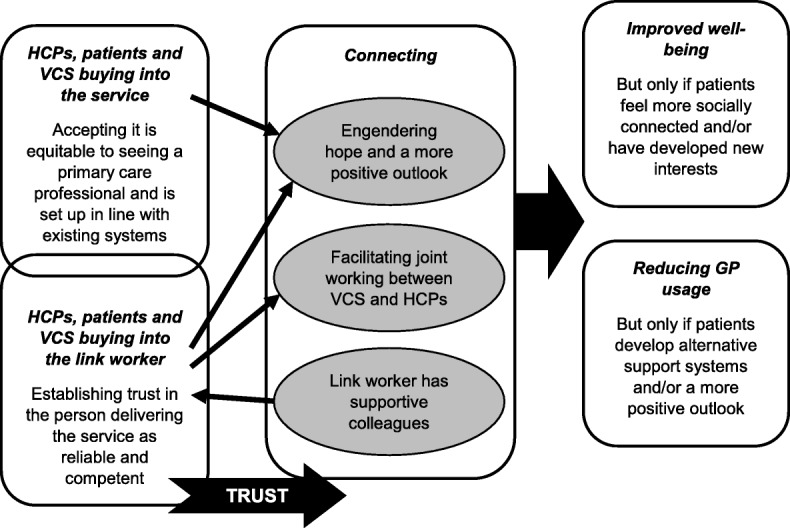
Our intermediate programme theory (prior to extending our understanding by drawing on existing theories)

Figure 4 brings together the broad concepts described above into an intermediate programme theory. It illustrates how ‘buy-in’ allows for connection. ‘Buy-in’ is required initially; otherwise, connections will not be made and cemented. Connection involves building and sustaining productive working relationships. In the figure, there is overlap between service and individual ‘buy-in’ because belief in someone delivering a social prescribing connector role may strengthen stakeholders’ belief in the service, or their ‘buy-in’ to the rationale behind the service prompts them to support an individual connector’s work. Within the programme theory, a cultural change in avenues considered acceptable for addressing health and well-being may be required from stakeholders for ‘buy-in’ to transpire. Hence, time and energy should be invested, upfront, in promoting the service to all key stakeholders as complementing medical care; otherwise, the development of trust and associated connections may be hindered.

### Contribution of existing theory

As noted in the methods section, we further refined and organised our understanding of social prescribing connector roles by drawing on two existing theories; they were selected from theories mentioned in reviewed documents that we felt were particularly pertinent in explaining key elements of our programme theory. Based on our interpretation of the data from the review, we inferred that through developing ‘buy-in’ and strong relational connections, link workers mobilise resources that come from being part of social networks. We propose that these networks then prompt patients to feel more able and willing to manage their own health goals. Therefore, the existing theories we drew upon were *social capital* and *patient activation*.

#### Social capital

Social capital refers to the resources accrued from connections [22]. Putnam [23] identified two key forms of social capital:

- Bonding—close-knit networks that produce feelings of solidarity and reciprocity. It involves links with like-minded people, with some form of shared identity.
- Bridging—these ties tend to be weaker, more fragile, with less emotional closeness, but can be useful for gaining information or developing a new perspective as it means being part of a heterogeneous grouping.

In terms of connector schemes, bonding social capital could occur when colleagues support each other with problem-solving and managing difficult emotions; trust is necessary for this situation for link workers to voice concerns to their peers. It also relates to the connection between patient and link worker; the latter takes time to get to know the former’s situation, develops with them a personalised action plan and, depending on the patient, offers emotional support. Such bonding social capital may make patients feel safe to open up to a link worker. However, bonding social capital can entail some form of exclusion, only benefitting those with access to a network/group. This is how providers in the VCS sometimes described their experience of trying to make links with HCPs. When trust between these two groups is lacking, the link worker takes on a bridging role, forging a closer relationship between the VCS and HCPs. Bridging social capital can also be identified when link workers connect patients to organisations or activities where they meet people from outside their direct social sphere. This enables them to gain information or a fresh perspective and to cultivate new skills.

Bourdieu [24] is another key writer on social capital. His work focused on resources derived by an individual from social networks to pursue their goals. For Bourdieu, such networks and their benefits do not occur naturally, but call for input (time and symbolic exchange); from this perspective, social capital represents an intentional process and ongoing investment in anticipation of future dividends [25]. This relates to the need for time and resources upfront in establishing social prescribing connector schemes as a new way of working within healthcare.

Carpiano [26] integrated conceptualisations of social capital from Putnam and Bourdieu into the following schema, which underpinned our understanding of how it relates to social prescribing connector roles:

- Structural antecedents—structural forces have implications for the type and strength of social ties that can be drawn upon. In social prescribing, a vibrant VCS is required. Furthermore, link workers need time to develop up-to-date knowledge of local, quality VCS provision and should be supported in this role through supervision and training. Another antecedent is the importance of consulting with key stakeholders to ensure that any service can fit into existing primary care systems and to overcome potential accessibility issues (e.g. due to transport or disability). In our review, there also seemed to be ‘personal antecedents’ (e.g. the patient being at a place in life when able to contemplate making change). Overall, this is about providing the right foundations for trust in link workers to blossom.
- Social cohesion—trust in link workers forms a basis for connections to be developed, from which social capital can transpire. This may occur incrementally. It covers the idea of link workers facilitating interactions between the VCS and HCPs; a better appreciation by HCPs of the former means those running VCS services are brought into conversations about health and well-being. It also relates to the time and consistency called for in interactions between a patient and link worker.
- Social capital—resources that stem from social connections. When working with a link worker, these resources may be cognitive (e.g. trusting others, appreciating community assets), psychological (e.g. self-confidence, self-control, belonging) and instrumental (e.g. having contacts to draw on for practical support or advice).
- Outcomes—developing social capital (in its various guises) could increase a patient’s sense of well-being. As individuals feel more socially included and self-confident through joining groups and receiving helpful outside advice, they become less reliant on their GP. Alternatively, a strong connection with the link worker means the patient feels safe to disclose difficulties that then need input from a GP.

#### Patient activation

Patient activation is defined as people’s confidence, motivation and ability (skills/knowledge) to manage their health [27]. Patient activation brings into focus attitudes and beliefs as well as behaviours and knowledge [28]. The problem, according to Hibbard [29], is many providers just give patients information without understanding where they are in terms of believing they can control their health situation. This may be unsuccessful in assisting individuals with low activation levels, as they can feel overwhelmed by and have limited confidence in managing their health [27]. It is argued that by tailoring an intervention to someone’s activation levels, they are more likely to encounter small successes, which propels them forward rather than leaving them deterred due to a lack of achievement [27].

A patient activation measure (PAM) has been developed to gauge how motivated and able someone is to manage their health [30, 31]. People identified as activated on this measure appear more likely to adopt healthy behaviours (e.g. diet and exercise) and to have less hospital use [27]. Intervention components linked to increasing patient activation scores include those that help with skills development, problem-solving, peer support or engender change in beliefs and social norms [32]. Link workers can cover these components (e.g. encouraging patients to think of assets and solutions to their problems when co-producing an action plan, linking them to networks that can foster connections). Through feeling more activated, a patient may be motivated to invest in self-care, prompting them to visit a GP for advice.

#### Bringing existing theories into our programme theory

From reviewed literature, we have used abductive reasoning (i.e. making judgements about which theories provide the best and simplest explanations) to detail how social capital and patient activation relate to social prescribing connector roles. We argue that gains from increased social capital include reduced isolation, feeling that life has meaning and having a support system that can be consulted for advice. This transformation prompts someone to be more activated in terms of their ‘internal readiness and capabilities to undertake health-promoting actions’ [33].

**Fig. 5:**
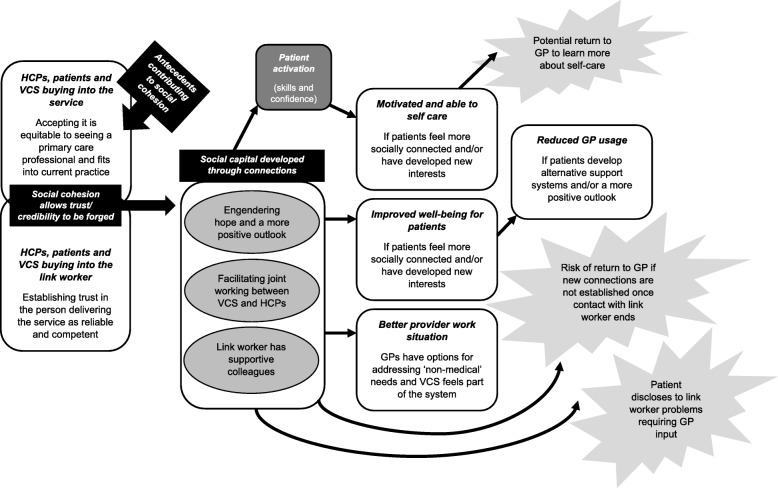
Revised final programme theory that draws on the reviewed literature and relevant substantive theory

Table 3 outlines a list of CMOCs we developed following further interpretation of our data through the theoretical lenses of social capital and patient activation (see Additional file 5: Final refined realist analysis, with supporting extracts from included papers). They build on the CMOCs we created for our intermediate programme theory (see Additional file 3: CMOCs for programme theory refinement, with data extracts). As illustrated in Fig. 5, our ‘final’ refined high-level programme theory shows that those likely to benefit from seeing a link worker are patients able to change their outlook on life, who can build and sustain their social capital. This may only happen when motivation and engagement are present. Feeling better connected and accruing resources from this (e.g. trusting in others, increased confidence, having people to access for assistance) may augment an individual’s activation to meet their health and well-being goals. Some patients are liable to find it relatively easy to make social contacts and move forwards. Others may require encouragement and direction from the link worker. It should be borne in mind that not all patients will be comfortable forging greater ties with outside groups or organisations. Hence, although accessing external support may enable patients to feel less alone or focused on their personal struggles, and/or bring hope of solutions to their problems, this will only work if someone deems that this is applicable to their circumstances. They also need to be able to access local assets that can address their needs; not all parts of the country may have a vibrant VCS to allow for this to happen. For the benefits from social prescribing to accrue, patients must be open to engaging with activities or accessing services (buy-in) and willing to make connections. Those who resist such external affiliation may not gain as much from seeing a link worker. There may be other patients reporting emotional solace from meeting with a link worker who still needs support from HCPs due to the complexity of their health condition.

**Table 3:**
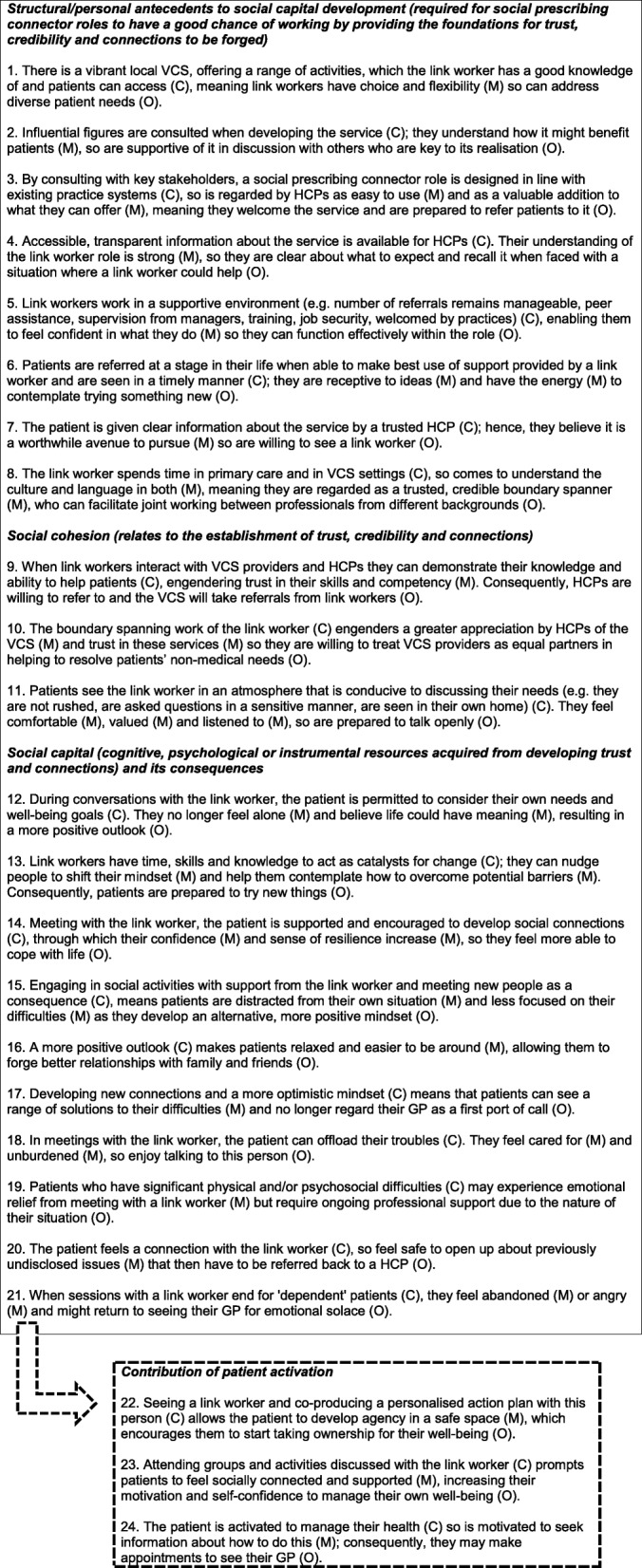
Refined realist analysis underpinned by a social capital and patient activation lens (see Additional file 5 for supporting data)

## Discussion

Social prescribing connector schemes are being established across England and in other healthcare systems. Policy documents, evaluations and papers included in our review suggest that desired outcomes associated with these roles are reduced GP workload and increased patient well-being. The remit of those acting in such a role includes supporting patients to address their ‘non-medical’ issues by connecting them to assets in the community (e.g. groups, organisations, charities, activities, events) that can help to improve their health and well-being. To the best of our knowledge, no previous systematic synthesis of existing literature, using a realist lens, has explored how this role can be implemented optimally. Other reviews have looked more at clinical navigation, rather than the use of local assets to address social determinants of health [34, 35], have not adopted a realist lens when working with data [36–38] or have not focused specially on connector schemes [39]. A realist review that explored social prescribing more broadly described link workers as essential but not enough, on their own, to ensure that desired outcomes are achieved [15]; other elements, as touched on in our review, need to be in place, such as a vibrant VCS, changing the medical model focus in some health settings, and a shift in the mindset of patients.

The realist review presented above, which drew on 118 documents, highlights the essential role of ‘buy-in’ and connections. These allow for trust to be forged, on several levels, resulting in social capital and patient activation. Our programme theory proposes that through meeting with a link worker, social capital (e.g. new skills, confidence and links) is developed, prompting patients to feel able to manage their health; individual activation levels are stimulated by engaging with social networks. Desired outcomes may then transpire, such as improved well-being and reduced reliance on a GP. However, there is potential for link workers to increase healthcare usage (see Fig. 5).

Link workers have received attention over recent months in England after the NHS long-term plan stated each PCN would receive funding for such a post [10]. Link workers do not have a specific qualification, although guidance on requisite skills/aptitude has been published [40]. It is a relatively wide-ranging post that necessitates good interactions with staff in PCNs and in the VCS. The guidance [40] calls for input from GPs and other staff in primary care to integrate link workers within multidisciplinary teams and to support individuals undertaking this role. Our review highlighted the need for primary care staff to be aware of the added value that this role offers; without feedback on how link workers have helped patients, staff may remain ambivalent and ‘buy-in’ be curtailed. If a link worker is serving several GP practices (as part of a PCN), it may be hard to establish a presence and to integrate fully, thereby jeopardising ‘buy-in’ to their individual skills and knowledge.

Being visible may be further hindered by the number of tasks link workers are expected to undertake, which include seeing and supporting patients, quality assessing local assets and engaging in relevant training [40]. In addition, they are required to develop new community groups when a gap is identified. This will help to bring together people with a similar need, leading to bonding social capital by uniting individuals facing comparable challenges. Bridging social capital may occur as link workers become part of multidisciplinary teams within primary care [4], thereby generating awareness of local assets among HCPs. At the same time, link workers have to bridge interactions between the VCS and HCPs [40].

Our programme theory proposes that by supporting people to develop their social capital, link workers can help to increase activation levels. Patient activation is identified by NHS England as a key measure for assessing link worker services within PCNs [41]. This reflects a broader policy commitment to prevent illness by tackling causes (e.g. loneliness, lifestyle choices, anxiety, low mood) and not just the symptoms of poor health [42]. To do so calls for a move beyond the traditional health and social care system by drawing on local assets and attending to social determinants of health; link workers can contribute to this aim, although wider systemic and cultural change is also required, with responsibility accepted across governmental departments (not just health and social care) [43].

### Strengths and limitations

We were systematic and transparent in the approach adopted to synthesise data, involving more than one reviewer in screening, inclusion of studies and data extraction. The CMOCs and programme theory were developed through regular team discussions; analysis encompassed input from people who varied in academic and clinical backgrounds. CMOCs were underpinned by data from reviewed documents and by two substantive theories. Regular consultation with stakeholders, including members of the public, helped to shape the findings, by assisting us with refining CMOCs.

Limitations include a reliance on existing literature. To develop our programme theory on how and why link workers help patients, we have had to make inferences based on a mix of data from included studies and existing theory. The plausibility of our inferences would be further strengthened by more primary data that specifically focussed on understanding the operation of these theories within the setting of connector schemes. Evidence on social prescribing, and link workers more specifically, is evolving. The methodological rigour of included papers was not always strong (e.g. a lack of clarity in how data were collected, low response rates, non-validated measurement tools). However, by triangulating qualitative and quantitative findings from across 118 documents, we believe that we can plausibly make the knowledge claims regarding the role of social capital in prompting people to feel connected and valued, and this contributing to a more active investment in health and self-care. It should be noted that such benefits may emerge if patients take up and engage in interactions with a link worker and feel that referral to such an individual is relevant to their perceived needs. The data we reviewed highlights that in some services, levels of attrition at the point of referral or after the first meeting with a link worker can be high; this emphasises the importance of ‘buy-in’ that formed part of our programme theory. We are aware that there is a long ‘implementation chain’ from someone engaging with a link worker to finally getting any benefits. There are potential gaps in this chain to benefits, which we have highlighted below as being areas that need additional research.

In our survey of all CCGs in England, the most common social prescribing connector role mentioned was for existing members of staff, usually receptionists, to act as care navigators [7], yet data on the role of receptionists in providing active signposting were less present in the literature we reviewed. Furthermore, there was a lack of literature on the potential of social prescribing connector schemes to produce sustained benefits in terms of patient well-being. These are areas for future research. In addition, there is a lack of robust evidence on how link workers impact on particular populations. Hence, future research could explore how link worker services affect specific patient groups (e.g. individuals with a learning disability, young carers, frail older people).

### Implications for practice and policy

Our review brings to the fore mechanisms that need to be ‘triggered’ to increase the prospect of desired outcomes from social prescribing connector schemes. It highlights contexts that mean these mechanisms are likely to be activated. Different iterations of the connector role attempt to achieve similar outcomes yet may be more or less successful in triggering requisite mechanisms depending on who delivers a service, in what environment and with what support. This has implications for countries like the UK, where social prescribing has become an established component of patient care, and other locations starting to introduce this complementary support for people’s ‘non-medical’ issues. When designing and implementing social prescribing connector schemes, areas for consideration include the following.

#### Getting ‘buy-in’ when delivered by a dedicated link worker

Patients may be wary about speaking to someone they do not know; how the messenger (e.g. HCP, written information) broaches seeing a link worker as an option should be given consideration; otherwise, there is a risk of it being rejected by patients. If a link worker is serving several practices (e.g. in a PCN), then waiting lists may increase; this could jeopardise ‘buy-in’ from patients and HCPs.

#### Getting ‘buy-in’ if using existing members of staff (e.g. receptionists)

‘Buy-in’ may be difficult to establish because of a perceived lack of status compromising their credibility. Locating care navigators within a GP practice means patients could see a mismatch between their attendance at a medical facility and being directed to ‘non-medical’ services. Clear communication to patients is therefore required about the care navigator role.

#### Establishing connections when delivered by a dedicated link worker

These individuals usually have time to spend with patients, often talking to them on several occasions. This allows them to understand someone’s needs and to coproduce a comprehensive action plan. They may even attend groups or organisations with a patient who lacks confidence. When there is a dedicated link worker in place, who has time to get to know people, the patient might enjoy talking through their problems. They may struggle when sessions with the link worker end and return to the GP as a familiar source of support. Consequently, ending contact with a link worker needs to be given attention from the outset.

#### Establishing connections when care navigators are existing members of staff

Time to develop rapport and trust, so patients feel able to open up about their needs, may be lacking when receptionists take on this role, preventing them from exploring in-depth someone’s difficulties. Patients may not take up recommendations if just told about them in a single conversation. Training in communication skills may be required so care navigators can help patients to express their needs. It would also seem prudent to give these members of staff clear points in the day when they move from one role to another, so they and others are clear when their care navigators’ work ends and their other duties recommence.

### Implications for research

Our final programme theory has highlighted areas warranting further investigation, including:

- Short term—how a link worker is best integrated into primary care so that ‘buy-in’ can be developed, how to recruit the ‘right’ people able to develop connections and what training/support they require to do so
- Longer term—sustainability of impact on patient well-being and cost-effectiveness of different types of connector roles

In relation to the substantive theories used, if social capital is core to social prescribing connector schemes, which then engenders patient activation, future research could explore the following:

- In a link worker service, when is bridging social capital necessary, and when is bonding social capital called for and for what purpose?
- Do different forms of connector roles generate different forms of social capital (e.g. bridging or bonding)?
- Does increased patient activation through interacting with a link worker lead to sustained improvements in outcomes? Do people need to reach a certain threshold in terms of activation to maintain improvements?

It should be noted that there were two topics extracted from included documents that we have not focused on in this review. One is the involvement of volunteers in supporting patients to use social prescribing. We feel this constitutes a separate review, and there are enough data to warrant such an endeavour. There were also documents that attempted to evaluate the cost-effectiveness of a social prescribing connector role. Again, this is an area warranting separate analysis, especially given the difficulty with how to assess cost-effectiveness in an intervention that has a range of consequences, with different stakeholders valuing different outcomes.

## Conclusion

Workload within primary care and changes to the VCS mean that GPs can struggle to maintain an up-to-date knowledge of local assets to meet the full range of support required to address individual health and well-being. This can hamper the delivery of personalised care. Social prescribing, and the introduction of link workers within PCNs, has formalised connecting patients to community resources to improve their daily living. As a complex intervention, social prescribing connector schemes should have a strong theoretical underpinning to account for how they work, for whom and why, to guide implementation on the ground. We have developed a programme theory for this purpose. It highlights that as a key component of social prescribing, link workers can help people to develop social capital (e.g. increase connections, develop new skills), enabling them to engage in activities that make life seem satisfying. In this respect, skilled, dedicated, knowledgeable link workers represent a precious commodity in primary care because of the social capital they can facilitate patients to build. Developing wider social networks prevents people from feeling isolated and exposes them to alternative perspectives and experiences of the world, giving life meaning. Activation levels may then increase; feeling more connected and in control could result in patients with the skills and confidence to manage their own health. We have outlined in this paper the areas to consider when establishing or running a social prescribing connector scheme to optimise outcomes, by raising awareness of relevant mechanisms and contexts required to trigger them.

## Supplementary information

**Additional file 1.** Figure of the initial programme theory.

**Additional file 2.** Search conducted on MEDLINE.

**Additional file 3.** CMOCs for programme theory refinement, with data extracts.

**Additional file 4.** Table of studies coded to develop CMOCs.

**Additional file 5.** Final, refined realist analysis, with data extracts.

## Data Availability

Datasets used in the review are available from the corresponding author upon request.

## Abbreviations

CMOCs: Context-mechanism-outcome configurations
GP: General practitioner
HCP: Healthcare professional
NHS: National Health Service
PCN: Primary care network
UK: United Kingdom
