# Supplementary material for: Supporting social prescribing in primary care by linking people to local assets: a realist review
Source: BMC Med. 2020 Mar 13;18:49. doi: 10.1186/s12916-020-1510-7 (PMC7068902; doi:10.1186/s12916-020-1510-7)
Supplement: Supplementary file 1 — Additional file 1. Figure of the initial programme theory. [file 12916_2020_1510_MOESM1_ESM.docx]

**Additional file 1: Initial programme theory ideas (March-April 2018)**

As a HCP, how far do I feel that the CN can handle this case? How much trust do I have in the CNs skills? Do I trust they will hand back to me if unable to manage?

Is the CN appropriately trained? What is the content of that training? Does it cover things like:

- Awareness of local services
- People skills
- Communication skills
- Cultural sensitivity

Is a one off meeting with CN enough? Depends on nature of the problem

(If appropriate) CN keeps in touch with patient

(If appropriate) CN refers patient on to another service for more support

(If appropriate) CN refers back to GP or HCP for additional support

**CN meets patient to explore how they could be helped with any social/ welfare/ emotional needs**

Self-refers to see a CN

Thinks they might benefit from seeing CN

Patient or relative hears about CNs

Decides to refer patient to CN

HCP works out patient has a social problem that CN could help with

Receptionist asks questions to see where best to direct the patient

(If appropriate) receptionist suggests other services than the GP

Patient goes to see a HCP at the surgery

Patient contacts the surgery and talks to a receptionist

***Abbreviations:***

***CN = care navigator***

***GP = general practitioner***

***HCP = healthcare professional***
