# Supplementary material for: Supporting social prescribing in primary care by linking people to local assets: a realist review
Source: BMC Med. 2020 Mar 13;18:49. doi: 10.1186/s12916-020-1510-7 (PMC7068902; doi:10.1186/s12916-020-1510-7)
Supplement: Supplementary file 2 — Additional file 2. Search conducted on MEDLINE. [file 12916_2020_1510_MOESM2_ESM.docx]

**Additional file 2: Search conducted on MEDLINE**

| [# ▲](http://ezproxy-prd.bodleian.ox.ac.uk:2081/sp-3.29.0b/ovidweb.cgi?&S=AMBDFPKJIDDDOAKDNCFKFGIBKMNIAA00&Sort+Sets=descending) | **Searches** | **Results** |
| --- | --- | --- |
| 1 | Patient Navigation/ | 451 |
| 2 | ((care or healthcare or community or outreach or patient?) adj5 navigator?).ti,ab. | 639 |
| 3 | (navigator? adj5 (program* or intervention* or service* or system?)).ti,ab. | 398 |
| 4 | ((care or healthcare or community or outreach or patient?) adj2 navigat*).ti,ab. | 1498 |
| 5 | (navigat* adj2 (program* or intervention* or service* or system?)).ti,ab. | 5381 |
| 6 | community health workers/ or medical receptionists/ | 4575 |
| 7 | (receptionist? or reception staff or reception personnel).ti,ab. | 484 |
| 8 | (health* adj (assistant? or aide? or advisor? or adviser? or advocate? or co-ordinator? or coordinator? or connector? or officer? or facilitator? or liaison or broker? or coach* or promoter?)).ti,ab. | 5003 |
| 9 | (community adj (assistant? or aide? or advisor? or adviser? or advocate? or co-ordinator? or coordinator? or connector? or officer? or facilitator? or liaison or broker? or coach* or promoter?)).ti,ab. | 639 |
| 10 | (link worker? or healthy living coach*).ti,ab. | 47 |
| 11 | 6 or 7 or 8 or 9 or 10 | 10366 |
| 12 | ((navigat* or sign post* or signpost* or path* or guided or guiding or refer*) adj5 (service? or system? or care? or healthcare or patient? or community* or support)).ti,ab. | 255325 |
| 13 | ((navigat* or sign post* or signpost* or path* or guided or guiding or refer*) adj5 (group? or club? or selfhelp* or self-help or education or learning or exercise? or physical activity)).ti,ab. | 55792 |
| 14 | social prescri*.ti,ab. | 50 |
| 15 | (navigat* or sign post* or signpost*).ti. | 10155 |
| 16 | 12 or 13 or 14 or 15 | 307530 |
| 17 | 11 and 16 | 535 |
| 18 | (active* adj3 (signpost* or sign post*)).ti,ab. | 5 |
| 19 | 1 or 2 or 3 or 4 or 5 or 17 or 18 | 7206 |
| 20 | limit 19 to "reviews (maximizes specificity)" | 127 |
| 21 | general practice/ or family practice/ | 71832 |
| 22 | general practitioners/ or physicians, family/ or physicians, primary care/ | 24357 |
| 23 | Primary Health Care/ | 67744 |
| 24 | Office Visits/ | 6410 |
| 25 | (ambulatory adj3 (care or setting? or facilit* or ward? or department? or service?)).ti,ab. | 15631 |
| 26 | ((general or family) adj2 (practi* or physician? or doctor?)).ti,ab. | 108826 |
| 27 | (primary care or primary health care or primary healthcare).ti,ab. | 114822 |
| 28 | (clinic? or visit?).ti,ab. | 426613 |
| 29 | ((health* or medical) adj2 (center? or centre?)).ti,ab. | 107995 |
| 30 | Community Health Services/ | 29798 |
| 31 | community.ti. | 119798 |
| 32 | (community adj3 (service? or care or health*)).ti,ab. | 63900 |
| 33 | 21 or 22 or 23 or 24 or 25 or 26 or 27 or 28 or 29 or 30 or 31 or 32 | 883157 |
| 34 | 19 and 33 | 1233 |
| 35 | 20 or 34 | 1325 |
| 36 | Developing Countries/ | 69931 |
| 37 | (Africa or Caribbean or West Indies or South America or Latin America or Central America).hw,ti,ab,cp. | 174638 |
| 38 | (Afghanistan or Albania or Algeria or Angola or American Samoa or Armenia or Armenian or Azerbaijan or Bangladesh or Benin or Byelarus or Byelorussian or Belarus or Belorussian or Belorussia or Belize or Bhutan or Bolivia or Bosnia or Herzegovina or Hercegovina or Botswana or Brazil or Brasil or Bulgaria or Burkina Faso or Burkina Fasso or Upper Volta or Burundi or Urundi or Cambodia or Khmer Republic or Kampuchea or Cameroon or Cameroons or Cameron or Camerons or Cape Verde or Central African Republic or Chad or China or Colombia or Comoros or Comoro Islands or Comores or Mayotte or Congo or Zaire or Costa Rica or Cote d'Ivoire or Ivory Coast or Cuba or Djibouti or French Somaliland or Dominica or Dominican Republic or East Timor or East Timur or Timor Leste or Ecuador or Egypt or United Arab Republic or El Salvador or Eritrea or Ethiopia or Fiji or Gabon or Gabonese Republic or Gambia or Gaza or Georgia Republic or Georgian Republic or Ghana or Gold Coast or Grenada or Guatemala or Guinea or Guinea-Bisau or Guam or Guiana or Guyana or Haiti or Honduras or India or Maldives or Indonesia or Iran or Iraq or Jamaica or Jordan or Kazakhstan or Kazakh or Kenya or Kiribati or Korea or Kosovo or Kyrgyzstan or Kirghizia or Kyrgyz Republic or Kirghiz or Kirgizstan or Lao PDR or Laos or Lebanon or Lesotho or Basutoland or Liberia or Libya or Macedonia or Madagascar or Malagasy Republic or Malaysia or Malaya or Malay or Sabah or Sarawak or Malawi or Nyasaland or Mali or Marshall Islands or Mauritania or Mauritius or Agalega Islands or Mexico or Micronesia or Middle East or Moldova or Moldovia or Moldovian or Mongolia or Montenegro or Morocco or Ifni or Mozambique or Myanmar or Myanma or Burma or Namibia or Nepal or Netherlands Antilles or Nicaragua or Niger or Nigeria or Pakistan or Palau or Palestine or Panama or Papua New Guinea or Paraguay or Peru or Philippines or Philipines or Phillipines or Phillippines or Romania or Rumania or Roumania or Rwanda or Ruanda or Saint Lucia or St Lucia or Saint Vincent or St Vincent or Grenadines or Samoa or Samoan Islands or Navigator Island or Navigator Islands or Sao Tome or Senegal or Serbia or Sierra Leone or Sri Lanka or Ceylon or Solomon Islands or Somalia or Sudan or Suriname or Surinam or Swaziland or Syria or Principe or South Sudan or Tajikistan or Tadzhikistan or Tadjikistan or Tadzhik or Tanzania or Thailand or Timor-Leste or Togo or Togolese Republic or Tonga or Tunisia or Turkey or Turkmenistan or Turkmen or Tuvalu or Uganda or Ukraine or Uzbekistan or Uzbek or Vanuatu or New Hebrides or Vietnam or Viet Nam or West Bank or Yemen or Zambia or Zimbabwe or Rhodesia).hw,ti,ab,cp. | 2320637 |
| 39 | ((developing or less* developed or under developed or underdeveloped or middle income or low* income or underserved or under served or deprived or poor*) adj (countr* or nation? or state? or population? or world)).ti,ab. | 83636 |
| 40 | ((developing or less* developed or under developed or underdeveloped or middle income or low* income) adj (economy or economies)).ti,ab. | 428 |
| 41 | (low* adj (gdp or gnp or gross domestic or gross national)).ti,ab. | 214 |
| 42 | (low adj3 middle adj3 countr*).ti,ab. | 10311 |
| 43 | (lmic or lmics or third world or lami countr*).ti,ab. | 5479 |
| 44 | transitional countr*.ti,ab. | 145 |
| 45 | 36 or 37 or 38 or 39 or 40 or 41 or 42 or 43 or 44 | 2478996 |
| 46 | 35 not 45 | 1023 |
