# Supplementary material for: Supporting social prescribing in primary care by linking people to local assets: a realist review
Source: BMC Med. 2020 Mar 13;18:49. doi: 10.1186/s12916-020-1510-7 (PMC7068902; doi:10.1186/s12916-020-1510-7)
Supplement: Supplementary file 3 — Additional file 3. CMOCs for programme theory refinement, with data extracts. [file 12916_2020_1510_MOESM3_ESM.docx]

| **Additional file 3: CMOCs developed to support initial programme theory refinement**  Realist analysis associated with legitimising the service  1.Support from influential figures (e.g. senior GPs, CCG, practice managers) (C) provides credibility to and validation of the service (M), so it is accepted by primary care staff as a viable alternative to medical care (O).  *“Having patronage of the CCG or health and wellbeing board was often an important step in signalling the value and importance of approaches and in garnering support.” (Gilburt et al., 2018: 40)*  *“Full engagement from senior management on a day to day basis resulted in the Navigator role being fully integrated into Practice and Pharmacy policies, systems and services.” (Deloitte, 2015b: 21)*  *“Getting anything like a full turn-out is a major challenge but the involvement of the GPs in this meeting provides a powerful symbolic endorsement of the work as something of real importance to the practice and could easily jump-start its acceptance by the whole practice by a year.” (McGregor et al., 2015: 18)*  2. Time is available when developing a service to consult with primary care staff (C); openness to and confidence in (M) the service is increased, so there is a readiness to integrate it into practices (O).  *“A slighter longer lead-in time, of approximately three months, would have been beneficial and allowed the service to test the referral system more fully, undertake more awareness-raising activity, and spend longer establishing a team culture and ways of working.” (OPM, 2017: 46)*  *“‘Implementation meetings’ were held every two weeks to discuss clinical processes and arrange operational procedures…GPs and other surgery staff, being present during these discussions enabled Community Navigation staff to develop a broader perspective on how the service fitted alongside other workstreams.” (Farenden et al., 2015: 38)*  *“When a model relies on the referrals, involvement and goodwill of clinical staff it is important that their views are sought at the very onset of commissioning to ensure buy-in and success.” (Wellbeing 4 U, 2018: 22)*  *“‘Co-design was done with all the partners sat around the table for a good length of time. We really got to the crux of what all the partners wanted to get out of the programme and the outcomes we wanted to see. And that helped to establish a shared vision.’” (Fullwood, 2018: 29)*  *“Although Altogether Better spent time engaging all groups of staff in the practice as a precursor to the work, it became clear that it worked less well in practices where the GPs and staff became disengaged and didn’t attend…planning meetings.” (Altogether Better, 2017: 20)*  3. HCPs receive clear information about the service (C); they regard LWs as a useful addition to primary care (M), so are willing to refer appropriate patients (O).  *“During the pilot, it became clear that providing direct feedback to GPs and practice staff either via the Navigator and/or more formally via a short periodic report was effective at encouraging a higher number of referrals from GPs as well as ensuring greater appropriateness of referral.” (Farenden et al., 2015: 42)*  *“We initially received a number of inappropriate referral but that has reduced significantly showing an increased understanding from the GP’s in regards to which of their patients will benefit the most from the service.” (Redbridge CVS, 2018: 8)*  *“The main reasons for GPs not referring were a lack of information about what the service offers/who is appropriate to refer and lack of time within the appointment to discuss this with their patients.” (Ferguson and Hogarth, 2018: 25)*  *“When exploring rates of referral, some interviewees suggested that some practices and individual clinicians didn't make full use of the Service due to lack of understanding about what Social Prescribing is and what it can offer patients.” (Dayson and Bennett, 2016: 5)*  *“From the implementation period it became evident that the need to educate all practice staff in the concept and impact of Social Prescribing is vital before a coordinator starts taking referrals. This improves the level of communication between health staff, patient and SP and helps ensure appropriate referrals are made.” (Wellbeing 4 U, 2018: 20)*  *“Health and care professionals identified that the most valuable aspect of the Community Navigator service was in helping them manage their caseloads. They saw the Navigators as essential in helping maintain a ‘flow’ of clients through the system.” (Barber, 2017: 37)*  *“‘…it’s really good as a clinician to be able to have somebody else that you can refer on to rather than just having to give them a prescription or something….(the SPHTs) need to have a good understanding of what is available out there you know, to be able to refer patients to other services.’” (White et al., 2010: 25)*  4. When the referral system is convenient (C), doctors refer to the service (O) because they perceive it is easy to do (M).  *“By ensuring the early set up of IT systems the SPC…could book patient appointments straightaway and understand the circumstances around why they were referred.” (Healthy Dialogues Ltd, 2018: 23)*  *“…98% of respondents to the survey of referrers felt that the referral process was straight forward. Many commented how easy the process was, particularly in Networks where they are able to refer by entering a code into EMIS or asking the reception team to book appointments directly.” (Ferguson and Hogarth, 2018: 40)*  *“The project staff have worked with practice staff to develop a 1 page referral form for GPs to use which can be accessed from their IT system and which automatically populates with the required information. The GP needs simply to insert the patient name and, if they choose, can add more information in a comments box.” (Baines, 2015: 20)*  *“…we needed to ensure our systems harmonised with those of each surgery…Some surgeries use a digital form that is completed and accessed on screen and some use a form that we created on paper and place this in a designated Navigator’s tray.” (Farenden et al., 2015: 41)*  5. Patients are referred at a stage in their life when able to make best use of support provided by a LW and are seen in a timely manner (C); they are receptive to ideas (M) and have the enthusiasm (M) and energy (M) to contemplate trying something new (O).  *“Regularly engaging with services was challenging, particularly for people whose condition fluctuated and those suffering from more than one health problem.” (Moffatt et al., 2017: 8)*  *“…proportions of both males and females referred but not assessed aged 18-39 were higher compared with patients who did attend for an assessment. It is possible that patients in this age group had lower levels of need or more chaotic lifestyles and therefore decided not to attend the PEP service.” (EMBED Health Consortium, 2016: 32)*  *“A number of patients referred had no intention of changing their behaviour and typically denied they had a problem. Even where patients acknowledged they ‘had a problem’ many did not have a desire to seriously want to address it.” (ERS Research and Consultancy; 2013: 18)*  *“101 patients of the 393 referred were not able to complete the Navigation journey. 71 did not attend the service at all, either because they were too ill, did not want the referral, were not feeling ready to think about solutions to problems or did not attend appointments made. 30 people began working with a Navigator but became unable to engage with the process for similar reasons.” (Farenden et al., 2015: 22)*  *“‘Some patients are just not ready or they have other bigger issues’ (SP coordinator)” (Carnes et al., 2015: 18)*  *“…some GPs will refer people who they feel are ready to take action to address some of the issues in their lives, given the right support. Others will refer people whom they feel the system is unable to help, hoping that the CLW approach will offer a solution to them.” (Innovation Unit, 2016: 14)*  *“Patients are seen within two weeks of referral which enabled them to address their issues or concerns quickly through voluntary and community channels.” (Healthy Dialogues Ltd, 2018: 24)*  *“There was also a considerable delay from referral date to beginning social prescribing activity, a feature which may have contributed to loss of enthusiasm and hence engagement.” (Loftus et al., 2017: 99)*  6. Patients are unclear about the service (C), perceiving referral as stigmatising (M), making them reluctant to see a LW (O).  *“For GPs, there was an initial challenge in getting patients to agree to engage with the Social Prescribing Service. This included ensuring that patients understood the purpose of the service and then accepted it as a viable complementary service to the medical support provided by the GPs.” (Dayson and Moss, 2017: 3)*  *“…GPs felt that engaging patients in the scheme was a barrier, partly because social prescribing is a new concept… ‘What’s diﬃcult is getting people to engage. I ﬁnd it hard explaining what it’s about. I haven’t come up with a nice easy phrase to say what it is. Partly because it’s so completely diﬀerent from what we tend to do.’” (Friedli et al., 2012: 25)*  *“Feedback from participating GPs’ practices indicates that there is some reluctance on the part of patients/clients to engage with the project. In part this was thought to be due to the original name for the project – Rugby Social Prescribing Project. This led some patients to believe that it was linked to social services and social workers which was off-putting.” (Baines, 2015: 23)*  *“…stigma attached to the non-medical needs of patients may act as a barrier to the uptake of initiatives beyond the traditional biomedical model of healthcare.” (Pescheny et al., 2018: 8)*  7. Patients have clear, accessible information about a SP connector scheme (C); it gives them confidence in the service as a viable means of addressing non-medical needs (M) and realistic expectations of how it can help (M), so they decide to try it (O).  *“One qualitative study reported that patients had poor knowledge of the service prior to attending their appointment with the link worker resulting in some feeling that the service did not meet their expectations.” (Bikerdike et al., 2017: 13)*  *“…some case study respondents reported not being sure what the programme entailed when initially agreeing to take part. It is suggested that information detailing what the programme offers could be provided in a leaflet format, which could be placed in areas such as General Practices and Pharmacies.” (Wigfield et al., 2015a: 8)*  *“Staff felt that it was important to ensure that the patient’s expectation of the service was realistic at an early juncture in the referral pathway, so that the outcomes of participation could match initial expectations: ‘I think it’s about the communication process with the patient and making sure that their expectations are right.’” (Woodall and South, 2005: 12)*  *“All of the patients attended the PSS with little or no knowledge of what to expect. This led ﬁve of the patients to form an expectation that the PSS was there to provide counselling. Consequently, patients became confused about the role of the PSS upon encountering them or, in two cases, disappointed that this role was not as expected, e.g. counselling.” (Faulkner, 2004: 44)*  *“Introducing a standard letter and information for all service users when they are first referred to the service, which explains that the service is a short-term intervention of 6-8 weeks. The letter also includes a central phone number, rather than their care navigator’s number. This has helped to manage service user expectations and reduce the number of phone calls that care navigators have to field.” (OPM, 2017: 10)*  8. When a service is situated within a medical setting (e.g. GP surgery) (C), some patients may struggle to see the coherence between what is offered and their perceived need (M), which means they risk rejecting support from a LW (O).  *“Many patients have an expectation, built up over many years, that their problems will be dealt with by the care system and clinical professionals. This can be hard to break down…” (Envoy Partnership, 2018: 36)*  *“…a few of the patient participants were deeply embedded in the medical model as a solution for their complaints. The GP was considered the most appropriate source for treatment, advice and cure.” (Brandling and House, 2007: 11)*  *“As SP, a non-clinical intervention, is implemented into a clinical setting, largely based on the medical model of health care, it may not be aligned with the expectations of some patients: ’Patients expect to be referred to an investigation, or a drug, not to SP!’ (GP 3)” (Pescheny et al., 2018: 7)*  *“‘I believe that having the signposters in a GP practice is not the best site…listening to feedback from those that have signposters in their surgery that patients just want to see a Nurse or GP…it compromises what the signposters may say. But outside of a GP setting it would work well and be greatly received.’” (RSM, 2017: 37)*  *“Lifelong habits of seeking help from the NHS do not change quickly. This includes the familiar presentation of physical symptoms that represent embodied psychosocial difficulties — a form of somatisation. The equally familiar response of bland reassurance from NHS staff to this form of somatisation is equally entrenched.” (Brandling and House, 2009: 455)*  9. The VCS is adequately resourced and informed about the service (C), so feels like a valued partner (M), consequently ‘buying-in’ to SP connector schemes as a concept (O).  *“The VCSE sector is involved from the start…Social prescribing relies on the capacity of small community groups to receive referrals and provide support.” (NHS England, 2019: 12)*  *“…the local voluntary and community sector (VCS) has benefitted from a catalytic investment in community level service provision, which has enabled small organisations without a track record in health service provision to access NHS funding…Some providers have been able to 'match' their Social Prescribing with income from other sources, to enhance their provision and improve the overall sustainability of their organisation.” (Dayson and Bashir, 2014: iii)*  *“Both primary care and voluntary organisation sectors must work together from the beginning of a scheme in order to establish how funding and sustainability can be managed…The VCSES themselves were keen to be involved in the development of social prescribing from the outset to ensure good working relationships, to manage capacity and funding issues…” (Brandling and House, 2007: 18-19)*  *“…there are indications that capacity within the sector may be insufficient to cope with rising demand, the high attendance at, and feedback from, the Social Prescribing Forums and Breakfast Events, as well as suggestions made by the VCS for more regular service updates, highlights the willingness of the local VCS to engage with social prescribing, which will continue to strengthen the service offer.” (Ferguson and Hogarth, 2018: 6)*  Realist analysis associated with belief in an individual LW  10. The LW has time to show an interest in the patient’s life, listening with a non-judgmental attitude (C). The patient feels valued and respected (M) and safe in the LW’s presence (M) and is therefore willing to open up about their needs (O).  *“The key active ingredient for the service is the time to develop the relationship with the client, holistically structured around a guided conversation.” (Darnton et al., 2018a: 4)*  *“Participants consistently reported feeling at ease and relaxed with their Link Worker, which enabled them to develop an open and trusting relationship.” (Moffatt et al., 2017: 6)*  *“For one interviewee the first home visit provided a sense of validation of her health conditions and housing problems that she had not received from other professionals…” (Aitken et al., 2017: 9)*  *“Patient 8 was relatively new to the practice and did not feel known by the GPs. This patient contrasted the experience with the doctors in the practice to with the CLP, who seems more willing or able to build a relationship with the patient, which implies he felt more valued…” (Mercer et al., 2017: 36)*  *“The patient will start to tell their full story, probably for the first time. The patient will feel overwhelmed and relieved all at once for having the opportunity to be listened to, and heard. We will discuss their priority issues and will make a plan on how best to move forward – discussing what support services may be available to them…” (NHS Wales, 2018: 1)*  *“All participants were asked, what is the most helpful thing they ‘do’ for their service users as an open- ended question and again it is clear that listening was the most important action that they could ‘do’ as it was mentioned by over a half of all participants (52%).” (National Association of Link Workers, 2019: 14)*  11. As the patient is able to open up (C), the LW can understand things from this individual’s perspective (M) and proposes personalised solutions to their problems (O).  *“The coordinator explores individual health and social needs, and then refers them to appropriate services according to their individual requirements.” (Palmer et al., 2017: 18)*  *“Navigators work one-to-one with patients, adopting a motivational interview technique ‘guided conversation’ to assess non-medical support needs. They work in a person-centred way to find solutions which fit priorities identified by the patient.” (Farenden et al., 2015: 10)*  *“Patients can talk about anything they like in their Wellbeing Review and staff are skilled at helping patients to drill down to the pertinent issues impacting on their health and wellbeing at the time…Working together they develop a personalised plan for wellbeing.” (Swift, 2017: 166)*  *“‘One of the reasons I think we get results is because we actually, spend the time with people to actually fully understand their needs, we spend that time getting to know the person inside out.’” (Barber, 2017: 39)*  12. Experiencing early, initial benefits from speaking to a LW (C) increases the trust the patient has in this individual (M), meaning they are willing to keep working towards making changes in their life (O).  *“Service users emphasised that the navigators’ person-centred approach facilitated feelings of trust, control, and readiness to reflect on their current circumstances and their non-medical needs. Service users reported that they engaged with further navigator appointments because they felt listened to and valued.” (Pescheny, 2018: 8)*  *“Spending time getting to know their clients and working from a more flexible approach allowed the Wellbeing Coordinators to develop trusting relationships, which ultimately seems to be critical factor for the on-going engagement of clients.” (Bunyan et al., 2017: 19)*  *“For clients who come to a CLW…it is often necessary to immediately put something in place, whether through attending an appointment together with Citizens Advice, taking them to the food bank or calling a housing officer to fix a broken boiler.” (Innovation Unit, 2016: 37)*  *“The relaxed personal approach of the SPC helped build good rapport and a trusting relationship with the patients…the SPCs ability to address some issues ‘there and then’ helped patients to take that first step towards supporting their recovery which was valued highly by patients.” (Healthy Dialogues Ltd, 2018: 24)*  13. The LW has time to spend in practices, so primary care staff get to know them as an individual and take strides to make them feel welcome (C); this helps with developing a connection (M) and trust (M) so the LW becomes a valued member of the team (O).  *“…some practice staff felt that the SPHT was not around enough and did not really feel like a full team member, others could not praise them enough: ‘It would be nice for her to be more integrated in the team. I know she likes it here and the staff you know, but in terms of….because she’s only here a little bit of the time….it’s a bit fly by night…‟ (BR22) “she fits in very well, she’s liked and people know her and recognise her around the place. ….she comes to our, we have a GP meeting on a Friday lunchtime…she’ll come and do a reminder to people...’ (BR20).” (White et al., 2010: 33)*  *“Champions need a certain amount of practical support to feel welcome – access to space (for themselves and posters), tea and coffee seem to be most important. Formal recognition of the champions by the practice is a powerful way to make them feel valued and recognised and places them as members of the team. But the most powerful form of recognition is when members of the General Practice team recognise the champions with as little as a ‘hello’ or a smile – and the GPs are particularly important.” (McGregor et al., 2015: 23)*  *“The full-time location of a CLP in the GP practice provided the opportunity for the CLP to become a trusted member of the GP team, where they could share information about community organisations.” (Skivington et al., 2018: 6)*  *“Maintaining a high and regular profile within the surgeries was found to help with referral numbers but this was hard to achieve with only two social prescribers.” (Bertotti et al., 2017: 26)*  *“At its best the social prescriber is seen as part of the primary care team, has a base in the practice, attends multi-disciplinary meetings to review patients and accesses and inputs to patients’ electronic health records.” (Liles and Darnton, 2017: 8)*  14. Through their interchanges with primary care and VCS staff, a LW demonstrates their competence and knowledge (e.g. at meetings and through feedback) (C); trust in this individual increases (M), resulting in HCPs’ willingness to refer people to the service and the VCS’ willingness to accept referrals (O).  *“…delivering feedback on participants’ progress encourages GP support for social prescribing.” (Bikerdike et al., 2017: 13)*  *“Clinicians who had experienced the Social Prescribing Service spoke very highly of the advisors, in terms of their knowledge, but also their skill in communicating with patients so that patients are at their ease. For example: ‘The sensitivity and the way she questioned, the way she teased-out information establishing what those needs were was just fantastic.’ (Clinician)” (Dayson and Bennett, 2016: 6)*  *“‘Her knowledge of all things knocks me over sometimes, you know, she has a vast knowledge of how she can help families. And she does it in a very unthreatening way.’” (Smith and Skivington, 2016: 13)*  *“Increases in the number of referrals are likely to occur where a feedback mechanism is in place as a reminder of the availability of the service and to highlight patients’ positive experiences.” (Bertotti et al., 2018: 240-241)*  *“The PCNs exhibited enthusiasm, a strong ethos of caring, and a passion for improving the health and well-being of their local population.” (Deloitte, 2015a: 2)*  *“Good feedback mechanisms are necessary to all potential referrers in order to close the loop and encourage more referral. For example, a link worker could contribute to patients’ notes, or inform the GP about improvements if that patient was referred to them from the surgery.” (Social Prescribing Network, 2016: 26)*  *“’You feel comfortable referring to them [care navigators] because you know they will get involved and they also know their limits so will tell you what they can or can’t do.’” (OPM, 2017: 17)*  *“Where Social Prescribers use EMIS for their case management, the referring health professional can review the consultation notes directly without additional feedback being required. In some Networks, or where GP’s have specifically requested it, feedback may be emailed or sent in a letter. Other means of feedback include as part of a monthly report on referrals received and verbal feedback given at clinical meetings.” (Ferguson and Hogarth, 2018: 16)*  Realist analysis associated with giving life meaning and inspiring hope  15. LWs are skilled in asking the right questions, in an encouraging way (C). This helps patients feel they have permission to contemplate their needs (M), meaning they start to see their situation in a more optimistic light (O).  *“…the social prescribing facilitator did not need a health qualification but a sound knowledge of the health and Third Sector systems. Someone with highly developed interpersonal, communication and networking skills, with a motivating and inspiring manner to encourage clients to make brave decisions or take up new opportunities.” (Brandling and House, 2007: 15)*  *“Knowledge of behaviour change and its relevance to health and wellbeing is a key requirement for the linkwork role and this should be explicitly captured.” (ERS Research and Consultancy, 2013: 17)*  *“Staff talked about the ‘wide range of approaches’ that they take to conversation – including: ‘breaking down what feels overwhelming, motivational interviewing, active listening, providing information - e.g. knowledge of rights’.” (Stocks-Rankin et al., 2018: 93)*  *“…the social prescribing service had enabled individuals to have a more positive and optimistic view of their life often through offering opportunities to engage in a range of hobbies and activities in the local community.” (Woodall et al., 2018: 1)*  *“In agreement with the quantitative data an improvement in wellbeing was reflected in the qualitative interviews with individuals describing feelings of optimism and a more positive outlook as a result of being referred…” (Bunyan et al., 2017: 5)*  *“The Co-ordinator visits people in their home and firstly tries to establish a rapport and understanding with the person, by listening to their story, and then supporting them to identify what really matters to them and what they want to achieve.” (Somerset CCG, 2016: 7)*  16. Continuity in the LW seen means patients trust this person to help them plan and prioritise (C), which reduces their cognitive load (M), so they feel more able to contemplate change (O).  *“Continuity is key in order to build trust and support the person.” (Community Works, 2017: 2)*  *“'[Link Worker] says, ‘I’m there, basically, any time,’ obviously within working hours, but she says, ‘Just phone me up if you need me, and any questions or anything.’ So basically I can see her as often as I want or as little as I want, but she likes me to keep her informed of anything happening, so she knows.' (P11, male, 45–49 years)” (Moffatt et al., 2017: 5)*  *“One CLP provided the example of a person with ten long-term conditions, who was taking about thirty different medications, as well as multiple social problems. The CLP explained that the only way to work with this person was to focus on the most immediate matters before moving on to more complex ones.” (Mercer et al., 2017: 47)*  *“During the initial consultation, where possible, Social Prescribers use MYCaW to facilitate clients to articulate their top two concerns and their feelings of general wellbeing…” (Ferguson and Hogarth, 2018: 16)*  *“‘It’s collaborative work. Not about me telling you how to live your life or what to do. Just two heads are better than one.’” (Bertotti et al., 2017: 2015: 19)*  *“’They invariably don’t come in with one problem. They come in with more than one and sometimes what they launch into ﬁrst of all, turns out not to be the main problem. They might come in with an emotional problem, but it turns out that they have a debt problem, which has caused the emotion. But they don’t tell you about the debt until you have dug it out of them.’” (Faulkner, 2004: 43)*  *“A critical aspect of these exchanges has been the additional time available to PICs to spend with clients in their own homes, and the continuity of their support over an extended period, thereby allowing trusting relationships to be established.” (Fullwood, 2018: 16-17)*  17. LWs have the capacity to not just tell people about available support but to offer solutions to potential barriers (C); hence, patients feel facilitated (M) and are more willing to take steps towards change (O).  *“Facilitated referral goes beyond simple signposting and describes a number of activities which might include; researching detailed information such as location, opening times, criteria, travel information…making appointments for a patient etc.” (Community Works, 2017: 3)*  *“The referral usually resulted in signposting to services but for some this extended to liaison between the patient and the organisation to facilitate access or even attending on the first occasion with the patient.” (Brandling and House, 2007: 11).*  *“‘When you are on your own, it’s difficult to just jump up and do new things, even if you really want to. It’s got a lot to do with confidence, as well. It’s that extra help that made a difference. She [the PIC] came with me the first time I went [to a crochet group]. She called me in the following weeks to encourage me to keep going, which I did and still do.’” (Fullwood, 2018: 18)*  *“Navigators believed that accompanying service users to first sessions, and helping them to build confidence, self-reliance, and eventually independence were crucial steps in determining the adherence of some service users…” (Pescheny et al., 2018: 8)*  *“The worker may accompany clients on their ﬁrst visit in cases where clients lack conﬁdence or require additional support.” (South et al., 2008: 312)*  *“Navigators helped clients to overcome barriers to access including; a lack of motivation or confidence to access services, dealing with administration, and challenges in securing reliable and affordable transport.” (Barber, 2017: 34)*  *“If a patient is lacking in confidence the Connector will go along with them to a service or activity until they are confident enough to go on their own.” (Dayson and Leather, 2018: NS)*  *“‘…she was always sort of reassuring and encouraging I was…when I ﬁrst began I was quite a…I rarely went out, I rarely did anything. It kind of gave me that boost to go and do things with the kids and I was always kind of worried about money. So she put me on to places that didn’t cost too much or were free sort of activities that sort of thing.’” (Friedli et al., 2012: 22)*  18. With support from the LW to engage in activities outside the home, patients become more connected to others in their local community (C); they feel less isolated and alone in their struggles (M), which can lift their mood (O).  *“Over the weeks, Martin became more and more confident as he was encouraged to try new activities and focus on solutions. His depression gradually lifted over the weeks.” (Community Works, 2017: 21)*  *“‘Best thing has been meeting new people and making friends. My mobile full up with names and numbers of friends before it was just family and doctor’s number.’*  *(service user)” (Bertotti et al., 2018: 239)*  *“The scheme also helped to raise awareness of the variety of support…available in the local community: ‘If I hadn’t of seen [the link worker]…I wouldn’t have got involved with [a service and service provider]. So she deﬁnitely helped me with that.’” (Friedli et al., 2012: 22)*  *“Those of the patient participants who could envisage themselves using such a service, felt that making the initial move to join a new group or seek help from a new or unknown source would be a challenge. They knew from experience that it takes courage to move away from known support and the home. A social prescribing facilitator was seen as a link between themselves and the unknown, someone who could make that leap a little less daunting.” (Brandling and House, 2007: 13)*  *“Clients discussed the ways in which the Service had changed how well they felt that they could cope with situations, in that they didn't feel isolated or alone with their problems any more.” (Dayson and Bennett, 2016: 21)*  *“These clients spoke passionately about how pursuing their interests had enabled them to widen their social circle and feel less lonely and isolated, as well as giving them a sense of purpose.” (Fullwood, 2018: 11)*  *“The analysis revealed that responses were significantly more positive at post stage than at baseline for all 3 statements:*   - *I am content with my friendships and relationships (z=-4.83, p<0.001) (n=313)* - *I have enough people I feel comfortable asking for help at any time (z=-4.32, p<0.001) (n=312)* - *My relationships are as satisfying as I would want them (z=-3.51, p<0.001) (n=309)” (Leeds Beckett University, 2018: 11)*   19. By meeting with the LW, patients are encouraged to engage with outside support and new activities (C). This can help to distract them or gives them an alternative focus (M), meaning they are less preoccupied with or stressed by their own circumstances (O).  *“Re-connecting with the world and renewed hope for the future were the two main patient experience themes that emerged.” (Bertotti et al., 2015: 6)*  *“Clients suggested that having someone sitting with them in order to discuss referral options meant it was far more likely that they would follow-up on referrals and make the changes suggested to them.” (Dayson and Bennett, 2016: 3)*  *“…the intervention enables the clients to engage in activities that supports them to manage the stress that their situation may be causing making them stronger and more resilient to continue.” (Redbridge CVS, 2018: 5)*  *“…when she was referred Mrs C didn’t expect to get anything out of Social Prescribing but has since realised that she now does not feel as isolated and was ‘just looking at four walls without the service’ and noted ‘while you’re here you don’t think about your health conditions, you just get on with it’". (Dayson and Bashir, 2014: 25)*  20. Making contact with local services and support (C) means patients feels less anxious or stressed (M), thereby improving their relationships with friends and family members (O).  *“There was a statistically significant decrease in GAD-7 Anxiety scores from baseline (M=15.39, SD=4.67) to three months after (M=7.21, SD=5.34), t (69) = 12.83, p= < 0.001. The mean decrease in GAD-7 Anxiety scores was 8.81 with a 95% confidence interval ranging from 6.901 to 9.442. The eta squared statistic (0.70) indicates a large effect.” (Kimberlee et al., 2014: 41)*  *“‘Once I started to see the Community Navigator, I didn’t have to keep asking my GP questions about other services as I knew that was all taken care of. There was a ripple effect throughout my whole family; my wife and child were better supported and cared for and my parents felt relieved. She’s helped me more than I can tell you. I wouldn’t have got this far without her.’” (Community Works, 2017: 21)*  *“‘Feeling more confident through working with the link worker has had a big impact on my relationship with my other half. He didn’t know how unhappy I was. Now I feel I can tell him what I need and have a night off from looking after the kids now and then.’” (Innovation Unit, 2016: 38)*  *“‘My husband says he can notice a difference when I’ve been out and done something rather than staying at home all day. It’s given us more to talk about as well.’ (Female client)” (Bunyan et al., 2017: 13)*  21. Through trying out suggested activities or attending relevant organisations (C) the patient’s skills and self-confidence increase (M), and they feel more in control of life (M), so take steps to improve their self-care (O).  *“In many cases ‘moving forward’ is not measured by hard indicators such as finding employment or an educational/training course, but by evidence of greater confidence or self-esteem or reducing isolation.” (Palmer et al., 2017: iii)*  *“‘I received DLA, income support and a budgeting loan. Though my health problems are the same [arthritis, back and joint problems] my stress has reduced immensely allowing me to cope a lot better.'” (Greasley and Small, 2002: 3)*  *“PEP referred the patient to the Financial Inclusion worker and recommended that she contact IAPT for counselling. PEP also referred her to a confidence course and linked her to community employment support…The patient now has a regular income, and has become more active. She has stated that she feels much more motivated now and that she feels she can “get back to being me” (feeling happier and confident in herself).” (EMBED Health Consortium, 2016: 102)*  *"Change in health-related behaviour and long term condition management was facilitated through the use of setting realistic, progressive and personalised goals…and social support.” (Moffatt et al., 2017: 9)*  *“Taking back some control over their lives was very important to the people interviewed. They all felt pleased with what they had achieved and had gained the confidence to carry on dealing better with the difficulties they faced.” (White et al., 2010: 16)*  Realist analysis associated with integrating health and community assets  22. LWs have scope within their job to get to know primary care staff and those working in the VCS (C); they learn and understand the culture of each (e.g. their specific language and structures) (M) so can act as a credible bridge between HCPs and the VCS (O).  *“CLPs themselves expect to take a proactive role in finding and making ties with local organisations and make themselves available as a liaison between community organisations and the general practice.” (NHS Scotland, 2016: 8)*  *“Link workers need a good knowledge and understanding of the local area and resources available to support people as well as good relationships between the people working in these community and voluntary sector organisations.” (Social Prescribing Network, 2016: 26)*  *“People who work in the health, social care and third sectors have very different training and organisational cultures; this can make partnerships challenging as they have different languages, values and measures of success.” (Nesta, 2013: 10)*  *“CLPs were seen as being able to facilitate a community organisation presence within GP practices…Prior to the implementation of the Links Worker Programme it was said to be difficult to engage with practices…lack of GP time, reception staff or other gatekeepers preventing access, attitudes within practices against engagement and organisational processes. They appreciated that CLPs understood their services and had the potential to educate practice staff on available community resources.” (Smith and Skivington, 2016: 2)*  *“To allow understanding of the voluntary and community sector, Wellbeing Coordinators discussed how they maintained a good working knowledge of the assets in the community through engaging directly with organisations or through ‘umbrella’ groups representing the voluntary and community sector…” (Woodall et al., 2015: 10)*  *“It was only after the linguistic analysis had been reported that Altogether Better felt able to articulate something that it had known all along – that the work for Practice Health Champions, local teams and Altogether Better itself lies at the boundary between the formal world of the NHS and the more informal world of both champions and most volunteering.” (McGregor et al., 2015: 16)*  23. LWs exhibit their knowledge of each sector to key stakeholders in meetings or in written feedback (C), fostering an appreciation of, respect for and trust in the VCS among HCPs and vice versa (M) bringing these groups closer together in their joint endeavour to improve patients’ quality of life (O).  *“…although organisational and professional identities can act as barriers to boundary-spanning, developing an understanding of and respect for the values, roles and responsibilities of different professions can also underpin successful integration…Mutual recognition and understanding of the skills and capabilities people bring to their work also plays an important role in determining how people experience their work within teams.” (Gilburt, 2016: 34-35)*  *“Individuals who occupy boundary spanning roles facilitate the communication and sharing of expertise, linking groups who might be separated in terms of location, division, or function.” (Hunt et al., 2016: 8)*  *“Partnership working between the Health and Voluntary & Community Sector has promoted a shared understanding of the differing approaches and methods used to achieve positive outcomes for patients and a growing number of GPs demonstrate trust in the Voluntary & Community Sector by referring their patients to the service.” (Farenden et al., 2015: 8)*  24. HCPs become aware of support for patients offered in the VCS through the LW (C). This makes them feel less alone or constrained when facing a patient who has non-medical problems (M), taking pressure off them in the workplace (O).  *“…the PCN role can enable GPs and nurses to focus more on managing complex care including medical care, where PCN provide continuity and spend longer, less pressurised time with patients and carers.” (Health Education England, 2016: 10)*  *“Using active signposting to safely reduce possibly avoidable appointments with GPs could lead to financial savings but in reality will free up time for GPs to focus on patients with more complex needs…” (Siddiqui et al., 2017: 2)*  *“Care navigators attending multidisciplinary team (MDT) meetings have facilitated this by developing links and increasing joint-working with other local services and professionals. MDTs have benefited from the input of care navigators as they offer ‘on the ground’ knowledge of the experiences and needs of service users. Care navigators also act as a source of knowledge on local organisations…” (OPM, 2017: 4)*  *“‘The LWL worker is taking some of the strain off me, so that I can specialise more on clinical need.’ (Alison, Community Matron).” (Andrew, 2016: 24)*  *“GPs expressed some relief that they had something in their toolkit to offer patients who presented with issues for which no clinical intervention was appropriate.” (Leyshon et al., 2015: 6)*  *“An added bonus may be the reduction of workload and more capacity to focus on medical problems. Hence far from being about shifting responsibilities and transferring so-called problem patients, we argue that social prescribing should be seen as one way of extending primary care through partnership working.” (South et al., 2008: 314)*  Realist analysis associated with supporting the supporter  25. When LWs can talk about the role and its difficulties with colleagues (C), it prevents them from feeling overwhelmed (M), and fosters resiliency (M), allowing them to focus on the patient they are trying to help and to provide person-centred support (O).  *“…team members share information and knowledge – it is important to meet…and reflect on challenging cases.” (Health Education England, 2016: 40)*  *“…provision of clinicians’ supervision for Social Prescribers in practices was felt to be necessary. It was felt that this would relieve some of the emotional responsibility Social Prescribers experience and assist them in overcoming the challenges associated with more complex cases.” (Ferguson and Hogarth, 2018: 46)*  *“‘…non urgent matters are shared at supervision including my own wellbeing, concerns about a client situation and support from my seniors, an extra pair of eyes to ensure that all needs are being addressed and all safeguarding concerns are met…’” (National Association of Link Workers, 2019: 20)*  *“A peer network to provide a chance to share lessons learned and insights with PICs working in other localities…Creating opportunities for reflective learning and strong feedback loops have been crucial in supporting continuous improvement and maximising success.” (Fullwood, 2018: 37)*  *“The CN role is an intensive one…[they] receive challenging disclosures from patients concerning their thoughts, feelings and the context of their lives. Often the Navigator is the only person a patient may have spoken to in detail about their situation in a long time, if at all. This necessitates a level of emotional and mental resilience…” (Farenden et al., 2015: 39)*  *“‘Need regular debriefing with a qualified counsellor as we are seeing negative depressed difficult people, this is essential to our wellbeing to keep going and to be able to do our best’ ‘Supervision, as the role has an emotional impact on my own wellbeing.’” (National Association of Link Workers, 2019: 20)*  26. Training is provided to LWs as required (C), giving them confidence in their ability (M) to support effectively the people they see (O).  *“Care navigators value access to training as this is fundamental for increasing skills to maintain expertise within their service” (Naick, 2018: 36)*  *“Mental health issues are common, we see people at the end of life, dementia, people with anxiety…We get some training but it depends on what is locally available, a lot of ‘in house’ training.” (Health Education England, 2016: 16)*  *“…PCNs were able to access the e-learning, although a few suggested that they had difficulty finding the time to complete the e-learning together with their daily duties, especially where Practice management was not engaged or fully aware of the PCN role.” (Deloitte, 2015b: 20)*  *“…co-ordinators are spending considerable time researching benefit entitlements for patients referred when an appropriate training course would help them to better understand the intricacies of the benefit system.” (Kimberlee, 2016: 41)*  *“Some of the things people tell us they'll do differently as a result of the training: Be able to proceed with more confidence.” (Conexus Health, 2018: NS)*  27. A LW’s caseload remains manageable (C), meaning they do not feel overwhelmed (M) so can think of creative, personalised solutions to individuals’ problems (O).  *“High levels of persistence, support and ﬂexibility are needed to engage patients with complex needs and enable them to overcome barriers to accessing support…” (Friedli et al., 2012: 7)*  *“Although CLPs have relative autonomy and ability to support patients at a very broad level, this is time-consuming and the demand for their services is high. Each of the CLPs talked about being extremely busy with supporting patients, who often have complex circumstances and are in contact in times of crisis.” (Smith and Skivington, 2016: 26)*  *“Recognising that simply giving information to patients about services results in low uptake, most SP schemes involve a navigator to provide personal support.” (Pescheny et al., 2018: 2)*  *“‘Pressures on resources and the capacity of care navigators have to be taken into consideration: For us its capacity because we can’t always react quickly, we’re covering a huge area (care navigator).’” (Naick, 2018: 37)*  28. When adequate statutory services are lacking or have long waiting lists, LWs may receive increasingly complex cases due to HCPs’ trust in their abilities (C); LWs feel overstretched and overwhelmed (M) and may then leave the service (O).  *“It was suggested that more GPs were starting to understand and trust the Social Prescribing Service, and therefore may be referring more cases of greater complexity. It was also suggested that the withdrawal/reduction of other support and welfare services had led to increased need in the community which is not being picked-up on elsewhere.” (Dayson and Bennett, 2016: 4)*  *“SPHTs were concerned about the number of referrals they received which they felt were inappropriate…In their view, some patients were being referred to them because the waiting times to see mental health staff were so long.” (White et al., 2010: 24)*  *“We have seen an increase in the numbers of very vulnerable individuals presenting to the service in comparison to those seen in 2016/17. This has had a significant impact on staff wellbeing which we started to explore early in 2017/18...” (NHS Wales, 2018: 2)*  *“The high referral numbers have, however, contributed to high staff turnover of Social Prescribers within the programme (about 40%). This is something which should be explored to ensure that with any expansion of the scheme referral numbers are kept to manageable levels for the service capacity.” (Ferguson and Hogarth, 2018: 18)*  “Navigators and clients both reported requiring greater levels of support from adult care or mental health services, and that the difficulties in securing this support made them reliant on continued support from the Community Navigator service. Navigators often saw themselves as fulfilling work that would have previously been done by adult care or mental health services.” (Barber, 2017: 47)  *“18% of Community Navigation cases were identified as complex, compared with 4% in 2014…Statutory services are stretched or tightening their eligibility criteria due to funding cuts. People who previously would have access to a key worker, social worker or CPN are being referred to Community Navigation instead...” (Impetus, 2017: 4)*  *“…care navigators are working with a considerable number of service users with multiple and complex needs who require a lot of support, and take up a lot of time. Feedback suggests that, at times, care navigators are being expected to do too much for service users, and that the service receives referrals which are more appropriate for social services.” (OPM, 2017: 13)*  29. When an individual LW leaves a service (C), because they have embodied and tacit local knowledge and relationships with key stakeholders (M), the service may be at risk of functioning less effectively (O).  *“Reliance on individual leaders and staff, as well as high staff turnover, has led to difficulties in sustaining individual roles and to a waning of support for boundary-spanning activities when staff moved on to different organisations or left altogether.” (Gilburt, 2016: 27)*  *“Retention is difficult. There is a significant loss of skill and wasted resource as new staff are repeatedly trained for these roles.” (Tavabie and Simms, 2017: 95)*  *“Lack of continuity of staff was also a concern of community organisation representatives. Although they occasionally talked about better awareness of their service among GP practice staff, facilitated by the CLPs, they did not expect their collaborative relationship with the GP practice to continue if the CLP was no longer in post. The CLP was seen as the ‘connector’, without which access routes would be closed…” (Skivington et al., 2018: 6)*  *“Professional stakeholders in Redbridge, Barking and Havering reported that staff turnover had been unsettling for a minority of clients who had built strong relationships with PICs who had left. As a result, some clients had decided not to continue to engage with the PICP, and the benefits they had experienced were compromised.” (Fullwood, 2018: 44)*  *“When a care navigator leaves, it not only reduces capacity and increases pressure within the team, but it also affects relationships and networks that the care navigator has built up with professionals and service users.” (OPM, 2017: 47)*  *“There has been considerable operational challenge, most notably the high staff turnover which has meant that the team has had to continually shift capacity…At the time of writing the team’s capacity was reduced by 4.5 wte care coordinators impacting on waiting times and leading to the possibility of delays in intervention.” (Ealing CCG, 2018: 18)*  *“The continuity of the SP programme…was affected when two of the navigators left…which resulted in the loss of links to key personnel within the CCG and GP practices, delaying the delivery of the SP programme.” (Pescheny et al., 2018b: 10)* |
| --- |
