# Supplementary material for: Supporting social prescribing in primary care by linking people to local assets: a realist review
Source: BMC Med. 2020 Mar 13;18:49. doi: 10.1186/s12916-020-1510-7 (PMC7068902; doi:10.1186/s12916-020-1510-7)
Supplement: Supplementary file 4 — Additional file 4. Table of studies coded to develop CMOCs. [file 12916_2020_1510_MOESM4_ESM.docx]

**Additional file 4: Table of studies that were coded to develop CMOCs**

| Authors | Year | Title - source | Study design/methods | Sample/setting | Objectives |
| --- | --- | --- | --- | --- | --- |
| Abel et al. | 2018 | ‘Reducing emergency hospital admissions: A population health complex intervention of an enhanced model of primary care and compassionate communities’ - British Journal of General Practice. | Case control study. Quantitative. Healthcare usage data. | One GP practice. | To evaluate a population health, complex intervention on population health improvement and reduction of emergency admissions to hospital. |
| Aitken et al. | 2017 | ‘Facework and trust in facilitating health focused housing interventions’ - Plus One. | Qualitative study. Interviews. | Occupants from 15 households with housing problems. | To explore service users’ experiences of an information and advice team developed to facilitate health-focussed housing interventions. |
| Altogether Better | 2017 | ‘Altogether Better  Creating a New Model of Community Centred Practice in Barnet’ - Evaluation Report produced for the London Borough of Harrow and the Joint Public Health Service for Harrow and Barnet by Altogether Better. | Evaluation. Mixed methods. Survey, demographic data, focus groups, interviews | 7 GP practices. | To learn from a review of the Community Centred Practice work developed by Altogether Better. |
| Andrew | 2016 | ‘Living Well Links Evaluation Report’ – Age UK Sunderland. | Evaluation. Mixed methods. Monitoring data, star measuring tool, case studies. | Living Well Links Service | To evaluate the impact of the service. |
| Baines | 2015 | ‘Rugby Social Prescribing Project ConnectWELL  Harnessing community capacity to improve health and wellbeing’ – Roundberry Projects. | Evaluation. Mixed methods. Routinely collected data, interviews. | Warwickshire Community and Voluntary Action. | To examine the client journey, project set up and processes, partnership working, impact on VCS, barriers and gaps, cost of involvement and resources. |
| Barber | 2017 | ‘Co-production in health and care services: an evaluation of the Community Navigator Service’ – MSc Dissertation (University of Hertfordshire). | Evaluation. Qualitative. Focus groups and interviews. | Clients, VCS representatives, Community Navigators, Health/social care staff. | To evaluate the extent to which a Community Navigator service is improving health and wellbeing and improving efficiency health/social services. To identify improvements that can be made to the service. |
| Bertotti et al. | 2015 | ‘Shine 2014 final report Social Prescribing: integrating GP and Community Assets for Health’ - City and Hackney Clinical Commissioning Group and University of East London. | Evaluation. Mixed methods. Questionnaires, monitoring data, data on healthcare usage, interviews, focus groups. | 23 GP practices. | To carry out an economic and process evaluation. |
| Bertotti et al. | 2017 | ‘The Social Prescribing Service  in the London Borough of Waltham Forest: Final evaluation report’ – University of East London. | Evaluation. Mixed methods. Questionnaires, data on healthcare usage, community activities and employment, interviews, focus groups. | Social prescribing service delivered by phone. | To assess the impact and cost-effectiveness of a social prescribing scheme and see how it integrates with other systems. |
| Bertotti et al. | 2018 | ‘A realist evaluation of social prescribing: an exploration into the context and mechanisms underpinning a pathway linking primary care with the voluntary sector’ - Primary Health Care Research & Development. | Realist evaluation. Mixed methods. Questionnaire, interviews, focus groups, learning events. | 23 GP practices. | To evaluate a social prescribing pilot in the areas of Hackney and City in London. |
| Best et al. | (missing) | ‘Piloting the benefits of a Social Prescribing service within GP Surgeries’ – Lowestoft Rising. | Report on pilot. Mixed methods. Questionnaires and data on number and type of referrals. | 3 GP practices. | To test the impact of the service and show how primary care can be delivered differently using community providers. |
| Bickerdike et al. | 2017 | ‘Social prescribing: less rhetoric and more reality. A systematic review of the evidence’ – BMJ Open. | Systematic review. Mixed methods. | Care navigation in the UK. | To summarise evidence for the effectiveness of social prescribing programmes relevant to the UK NHS setting. |
| Brandling and House | 2007 | ‘Investigation into the feasibility of a social prescribing service in primary care: a pilot project’ – University of Bath. | Report. Qualitative. Interviews. | 3 GP practices. | To explore the feasibility of developing a social prescribing service and to develop a business case. |
| Brandling and House | 2009 | ‘Social prescribing in general practice: adding meaning to medicine’ – British Journal of General Practice. | Commentary paper. | N/A | To describe and debate the role of social prescribing within primary care. |
| Brownlee and Jenkins | 2018 | ‘Prescribing social activities to lonely people prompts ethical questions for GPs’ – The Conversation. | Commentary paper. | N/A | To consider the moral and ethical issues associated with social prescribing. |
| Bunyan et al. | 2017 | ‘Evaluation of connect for health: Interim report v.1: Initial findings’ – Leeds Beckett University. | Evaluation. Mixed methods. Interviews, focus groups, questionnaires. | Not clear | To explore the effectiveness of the service. |
| Carnes et al. | 2015 | ‘City and Hackney Social Prescribing Service: Evaluation Report’ - QMUL/Barts, London School of Medicine and Dentistry, University East London. | Evaluation. Mixed methods. Questionnaire, data on activity, healthcare usage, interviews, focus groups, observations. | 22 GP practices and an adult community rehabilitation team. | To explore the impact of the social prescribing project on patients, primary care team's awareness of community resources and cost of service. |
| Carnes et al. | 2017 | ‘The impact of a social prescribing service on patients in primary care: a mixed methods evaluation’ – BMC Health Services Research. | Matched control group study. Mixed methods. Questionnaire, data on healthcare usage, monitoring data, interviews. | 22 GP practices. | To evaluate the effect of the service on people referred and the implementation of the service from patients' perspective. |
| Central London CCG | 2018 | ‘Care Coordination Service Patient Feedback October 2018’ – Care co-ordination service. | Report. Quantitative. Questionnaire. | A care coordination service. | To provide patient feedback on the service. |
| Clarke et al. | 1992 | ‘Social intervention and the elderly: A randomized controlled trial’ – American Journal of Epidemiology. | RCT. | 12 GP practices. | To determine the effects of a social intervention on mortality and morbidity. |
| Collins and Flexen | 2017 | ‘Barton Social Prescribing and Health Living Centre’ - Bury Knowle Health Centre. | Report. Mixed methods. Case studies and routinely collected data. | 2 healthy living centres. | To summarise progress of social prescribing in this area. |
| Community Works | 2017 | ‘Social Prescribing Extended Pilot Interim Monitoring Report March 2017’ – Community Works, Possibility People, Impetus Community Navigation. | Evaluation. Mixed methods. Questionnaire, routinely collected data and case studies. | 308 people referred to care navigators. | To evaluate how far service outcome indicators are met. |
| Conexus Healthcare | 2018 | ‘Care Navigation Training & Consultancy: Delivering results nationwide’ – Conexus Healthcare. | Report. Quantitative. Questionnaire (with some open-ended questions). | Worked with about 1,100 GP practices. | To report on outcomes from training of staff to be care navigators. |
| Darnton et al. | 2018 | ‘Independent evaluation of the North East Hampshire and Farnham Vanguard  Making Connections Service’ – Wessex Academic Health Science Network. | Evaluation. Mixed methods. Questionnaire, interviews, economic evaluation. | 595 people referred to service. Data collected from 262 at baseline and 162 after being supported. | To evaluate the Making Connections service. |
| Darnton et al. | 2018 | ‘Independent evaluation of Care Navigators on the Isle of Wight’ - Wessex Academic Health Science Network. | Evaluation. Mixed methods. Questionnaires, interviews, case studies. | 16 GP practices. | To evaluate the care navigator service. |
| Dayson et al. | 2013 | ‘From dependence to independence: Emerging lessons from the Rotherham Social Prescribing project’ – Sheffield Hallam University. | Evaluation. Mixed methods. Monitoring data from VCS, data on healthcare usage, interviews, case studies. | 28 GP practices. | To assess the impact of a social prescribing pilot, whether it has reached its aims, cost-benefits, effectiveness to develop a business case. |
| Dayson and Bashir | 2014 | ‘The social and economic impact of the Rotherham Social Prescribing project: Main evaluation report’ – Sheffield Hallam University. | Evaluation. Mixed methods. Routinely collected data, data on healthcare usage, interviews, case studies, questionnaires. | 29 GP practices. | To assess the impact, cost-benefits and effectiveness of service delivery model, to develop a business case. |
| Dayson and Bennett | 2016 | ‘Evaluation of Doncaster Social Prescribing service: Understanding outcomes and impact’ – Sheffield Hallam University. | Evaluation. Mixed methods. Questionnaires, interviews. | Delivered by a housing association and a voluntary and community sector support organisation. | To assess the implementation, outcomes and impact of a social prescribing service. |
| Dayson and Moss | 2017 | ‘The Rotherham Social Prescribing Service for People with Long-term Conditions: A GP Perspective’ – Sheffield Hallam University. | Report. Qualitative. Interviews. | 1 GP practice. | To explore the benefits and challenges of social prescribing from the perspective of GPs. |
| Dayson and Leather | 2018 | ‘Evaluation of HALE Community Connectors Social Prescribing service 2017’ – Sheffield Hallam University. | Evaluation. Quantitative. Pre-post questionnaires. | 703 referrals (from GP) but didn't capture data on all of these. | To evaluate the early stages of delivering a service in Bradford. |
| Deloitte et al. | 2015 | ‘The Primary Care Navigator programme for dementia: Benefits of alternative working models’ – Deloitte Centre for Health Solutions. | Case study. Qualitative. Questionnaires, observations and visit discussions with staff, interviews. | 1 GP practice and 1 social enterprise. | To consider how training and the primary care navigator role has had an impact. |
| Deloitte et al. | 2015 | ‘Primary Care Navigator training programme for dementia Evaluation of its impact’ - Deloitte Centre for Health Solutions. | Evaluation. Mixed methods. Questionnaires, interviews. | 20 GP practices, 20 pharmacies. | To measure the effectiveness of training and implementing a primary care navigator training programme and to identify enablers and barriers to its success. |
| Dewan et al. | 2014 | ‘A service evaluation of the feasibility of a community based consultant and stroke navigator review of health and social care needs in stroke survivors 6 weeks after hospital discharge’ – Clinical Medicine. | Evaluation. Pre-post study. Questionnaires and clinical data. | Patients who had a stroke. Home visits. | To present the concept of consultant-led community stroke follow up and outline initial ﬁndings from a feasibility point of view. |
| Dixon et al. | 2005 | ‘A care workers’ revolution?’ – Community Care. | Commentary paper. | N/A | To consider the green paper and its impact on the social care workforce. |
| Dudley CCG | 2018 | ‘Integrated Plus Feedback survey 2018 (for GPs and health clinicians)’ – Integrated Plus. | Report. Quantitative. Questionnaire. | 55 GPs and health clinicians. | To gather feedback from healthcare professionals on a social prescribing intervention. |
| Ealing CCG | 2018 | ‘Ealing CCG Care Coordination Summary Report’ – Ealing CCG. | Report. Mixed methods. Questionnaire, case studies, interviews. | 7 GP networks including 76 practices | To present quantitative data and qualitative feedback on a care coordination service. |
| Edwards | 2018 | ‘RBWM and WAM CCG Social Prescribing update’ - Better Care Fund Board Meeting, East Berkshire CCG. | Report to a board. Quantitative. Questionnaires. | 20 GP practices. | To update on the progress of a social prescribing service. |
| EMBED Health Consortium | 2016 | ‘Patient Empowerment Project Final Evaluation Report’ - Leeds West CCG. | Evaluation. Mixed methods. Routinely collected data, questionnaires, case studies, information from patient medical records (e.g. HbA1c, blood pressure). | 37 GP practices | To analyse data on the service's aims and to discuss its impact. |
| Envoy Partnership | 2018 | ‘Self-Care Social Prescribing: Social return on investment’ - Kensington & Chelsea Social Council and NHS West London CCG. | Social return on investment evaluation. Mixed methods. Questionnaires, routinely collected data, interviews, case studies. | One Clinical Commissioning Group. | To analyse a self-care programme. |
| ERS Research and Consultancy | 2013 | ‘Newcastle Social Prescribing Project: Final Report’ - www.ers.org.uk | Evaluation. Mixed methods. Consultation with key stakeholders, interviews, documentary analysis (e.g. job descriptions and patient criteria). | 6 GP practices. | To assess the project's impact and achievements, and to document lessons learnt. |
| Farenden et al. | 2015 | ‘Community Navigation in Brighton & Hove: Evaluation of a social prescribing pilot’ - Brighton and Hove Impetus. | Evaluation. Mixed methods. Interviews, questionnaires. | 16 GP practices. | To evaluate a 12 month pilot. |
| Faulkner | 2004 | ‘Supporting the psychosocial needs of patients in general practice: the role of a voluntary referral service’ - Patient Education and Counseling. | Qualitative study. Interviews. | One GP practice. | To describe key features of a patient support service, its perceived effectiveness and barriers to effective service provision. |
| Ferguson and Hogarth | 2018 | ‘Social Prescribing in Tower Hamlets: Evaluation of Borough-wide Roll-out’ - Tower Hamlets Together. | Evaluation. Mixed methods. Healthcare usage data, interviews, questionnaires, focus groups, case studies, | 37 GP practices | To describe and evaluate how well the programme is meeting its objectives, and making recommendations for service development. |
| Friedli et al. | 2012 | ‘Evaluation of Dundee Equally Well Sources of Support: Social Prescribing in Maryﬁeld: Evaluation Report Four’ - Equally Well in Dundee. | Evaluation. Mixed methods. Data on GP prescriptions, questionnaires, interviews, link worker reflections, focus groups(?). | One medical centre. | To explore the feasibility of a social prescribing service in an area of deprivation. |
| Fullwood | 2018 | ‘Blended evaluation of Phase 2 of the Age UK Personalised Integrated Care Programme: Final evaluation report’ - Understanding Value Ltd. | Evaluation. Mixed methods. Routinely collected data, questionnaires, case studies, documentary analysis (minutes from meetings and report to funders). | 8 local health care partnerships across England. | To evaluate Age UK's Personalised Integrated Care programme. |
| Gilburt | 2016 | ‘Volunteering in general practice: Opportunities and insights’ – The King’s Fund. | Literature review. | Papers looking at roles to improve integrated care. | To review literature on boundary-spanning roles to support integrated care. |
| Gilburt et al. | 2018 | ‘Supporting integration through new roles and working across boundaries’ – The King’s Fund. | Scoping review from which case studies were located. | Documents shedding light on how volunteers contribute to the work and role of general practice. | To scope the literature to identify ways in which volunteers contribute to general practice. |
| Goodwin and Hendrick | 2016 | ‘Physiotherapy as a ﬁrst point of contact in general practice: a solution to a growing problem?’ - Primary Health Care Research & Development. | Pre-post study. Quantitative. Questionnaires, data from medical records. | Physiotherapy service. | To evaluate the clinical effectiveness, patient satisfaction and economic efﬁcacy of a physiotherapy service providing musculoskeletal care, as an alternative to GP care. |
| Grant et al. | 2000 | ‘A randomised controlled trial and economic evaluation of a referrals facilitator between primary care and the voluntary sector’ – British Medical Journal. | RCT. | 26 GP practices. | To compare outcome and resource utilisation among those referred to a referrals facilitator to those receiving routine GP care. |
| Grayer et al. | 2005 | ‘A graduate primary care mental health worker pilot study: facilitating access to voluntary and community sector services. A description of the ‘Community Link Service’’ - Primary Care Mental Health. | Pre-post study. Quantitative. Monitoring data, questionnaires. | 13 GP practices. | To assess the feasibility and acceptability of a pilot scheme - graduate primary care mental health workers who had limited training - in facilitating access to the VCS. |
| Grayer et al. | 2008 | ‘Facilitating access to voluntary and community services for patients with psychosocial problems: a before-after evaluation’ – BMC Family Practice. | Pre-post study. Quantitative. Questionnaires and data on healthcare usage. | 13 GP practices. | To evaluate the acceptability and effectiveness of graduate primary care mental health workers facilitating access to VCS services. |
| Greasley and Small | 2002 | ‘With poverty and ill-health inextricably linked, where better to give welfare advice than in GP practices?’ – Health Service Journal. | Descriptive paper. | 31 GP practices. | To describe how welfare advice was placed within GP practices. |
| Green and Ellerby | 2017 | ‘The social and economic impact of the integrated plus service: Main evaluation report’ - Dudley CVS | Evaluation. Mixed methods. Monitoring data, patient review data, interviews, case studies, questionnaire, healthcare usage data, discussions with staff. | 46 GP practices. | To provide information on the progress, learning and socio-economic impact of the service. |
| Greenwich CCG | 2014 | ‘Report on integrated care in Greenwich’ – www.greenwichccg.nhs.uk | Report. Provides an evaluation framework. | 14 GP practices. | To summarise progress towards delivering co-ordinated care. |
| Haggart et al. | 2018 | ‘Age UK Personalised Integrated Care Programme Learning Report’ - Dartford, Gravesham and Swanley CCG and Swale CCG. | Learning report. Mixed methods. Routinely collected data, questionnaires, case studies, cost effectiveness analysis. | 15 GP practices. | To reflect on the programme's success in achieving its aims and to consider lessons learnt. |
| Health Education England | 2016 | ‘Care Navigation: A Competency Framework’ – www.hee.nhs.uk | Policy document. Mixed methods. Literature review, interviews, focus groups, online consultations, documentary analysis. | Care navigation in the UK. | To describe a core, common set of competencies for care navigators. |
| Health Education England | 2016 | ‘Social prescribing at a glance North West England’ - www.nw.hee.nhs.uk | Report. Mixed methods. Literature review, case studies, round table event. | Social prescribing activities in north west England. | To encourage wider understanding and exploration of the benefits of social prescribing to promote wellbeing. |
| Healthwatch Stoke-on-Trent | 2018 | ‘Care navigation – phase 1: Patient experience’ – Healthwatch Stoke-on-Trent. | Patient experience report. Mixed methods. Survey and spoke to people. | 9 GP practices. | To understand patients' experiences of signposting to a GP receptionist. |
| Healthy Dialogues Ltd | 2018 | ‘Evaluation of the East Merton Social Prescribing Pilot’ – Merton CCG. | Evaluation. Healthcare usage data, questionnaire, interviews, focus groups. | 2 GP practices. | To review how effective the social prescribing pilot is at improving health and well-being and reducing GP workload. |
| Hunt et al. | 2016 | ‘The role of boundary spanners in delivering collaborative care: a process evaluation’ – BMC Family Practice. | Process evaluation. Mixed methods. Questionnaire, interviews, focus group. | 5 GP practices and a Community Mental Health Team. | To understand the Community and Physical Health Co-ordinator role as a boundary spanner and how it was operationalised alongside MDT meetings. |
| Impetus | 2017 | ‘Social prescribing in Brighton & Hove: Interim evaluation & service update’ - Brighton & Hove Impetus. | Evaluation. Quantitative. Questionnaire and data on service use. | 30 GP practices. | To chart the progress of a community navigator service. |
| Impetus | 2017 | ‘Impact report 2016-2017’ - Brighton & Hove Impetus. | Report. Unclear methods. | A single charity. | To report on the work over a one year period of a charity that aims to connect people to reduce isolation and improve wellbeing. |
| Innovation Unit | 2016 | ‘Wigan Community Link Worker Service Evaluation’ – Innovation Unit. | Evaluation. Mixed methods. Routinely collected data, case studies, provider reports, stakeholder workshops, interviews, | 63 GP practices eventually - started with 11. | To understand how the service is working, who is using it, what difference it makes to clients and referring services. |
| Kimberlee | 2016 | ‘Gloucestershire Clinical Commissioning Group Social Prescribing Service: Evaluation Report’ – University of the West of England. | Evaluation. Mixed methods. Monitoring data, questionnaires, focus groups, interviews. | 81 GP practices (since March 2016). | To evaluate the effectiveness of an expanded social prescribing pilot. |
| Kimberlee et al. | 2014 | ‘Measuring the economic impact of Wellspring Healthy Living Centre's Social Prescribing Wellbeing Programme for low level mental health issues encountered by GP services’ – University of the West of England. | Evaluation. Mixed methods. Questionnaires, focus groups, interviews, healthcare usage data, routinely collected data. | Wellspring Healthy Living Centre | To evaluate a social prescribing service and measure its social and economic impact. |
| Kochane et al. | 2016 | ‘Community and Care Coordinator Project’ – Clinical Assurance Panel, Shropshire CCG. | Report for clinical assurance panel. Quantitative. Activity returns from practices, questionnaires, | 39 practices had a Community Care Co-ordinator at the time of evaluation. | To consider disinvestment and its potential impact on the health service. |
| Lambeth CCG | 2018 | ‘Freedom of information request response’ – Lambeth CCG | Freedom of Information request. Questionnaire. | One CCG. | To understand how care navigation was being implemented in the area. |
| Leeds Beckett University | 2018 | ‘Evaluation of Connect for Health: Quantitative Summary Report’ – Leeds Beckett University. | Evaluation. Quantitative. Pre-post questionnaires. | Unclear. | To evaluate the service. |
| Leveaux et al. | 2012 | ‘Moving in the right direction’ – Health Service Journal. | Descriptive paper. | N/A | To describe the introduction of care navigators into London. |
| Leyshon et al. | 2015 | ‘Living Well Penwith Pioneer: How does change happen? A qualitative process evaluation’ – University of Exeter. | Evaluation. Qualitative. Interviews, focus groups, observations, participatory tea parties. | Living Well service. | To explore processes through which Living Well, as a philosophy, has been operationalised, to examine how change was achieved, to understand lessons that can be learnt. |
| Liles and Darnton | 2017 | ‘Social Prescribing in Wessex Executive Summary: Understanding its impact and supporting spread’ – Wessex Academic Health Science Network and R-Outcomes. | Summary of outcomes from several services. Mixed methods. Questionnaires, case studies, interviews, data on service usage. | 8 social prescribing services. | To describe attempts to understand and evaluate social prescribing services in Wessex. |
| Loftus et al. | 2017 | ‘Impact of social prescribing on general practice workload and polypharmacy’ – Public Health. | Quantitative study. Pre-post. Data on healthcare usage and medication. | One GP practice | To investigate whether social prescribing activity influenced GP workload and polypharmacy. |
| Maughan et al. | 2016 | ‘Primary-care-based social prescribing for mental health: an analysis of ﬁnancial and environmental sustainability’ - Primary Health Care Research & Development. | Case-control study. Quantitative. Data on healthcare usage. | Connect project - run by Mind. | To assess the effects of a social prescribing service on healthcare usage, focusing on economic and environmental costs (carbon footprint). |
| McGregor et al. | 2015 | ‘Altogether Better Working Together to Create Healthier People and Communities  Bringing citizens and services together in new conversations’ – Altogether Better. | Report. Mixed methods. Interviews, focus groups, linguistic analysis, questionnaires, data on training. | 3 GP practices initially. | To report on the impact of the programme on people's mental well-being and the system. |
| Mental Health Foundation | 2013 | ‘Crossing Boundaries  Improving integrated care for people with mental health problems’ – Mental Health Foundation. | Report. Mixed methods. Literature search, expert attended seminars, call for evidence. | 31 experts attended the seminars and 1200 responses to call for evidence. | To identify good practice, generate discussion and draw key messages about integrated health for people with mental health problems. |
| Mercer et al. | 2017 | ‘Evaluation of the Glasgow ‘Deep End’ Links Worker Programme’ – NHS Scotland. | Quasi experimental study and process evaluation. Mixed methods. Questionnaires, interviews, focus groups, healthcare usage data. | 7 intervention GP practices and 8 controls. | To assess the implementation and impact of a link workers programme at a patient, practice and community level. |
| Moffatt et al. | 2017 | ‘Link Worker social prescribing to improve health and well-being for people with long-term conditions: qualitative study of service user perceptions’ – BMJ Open. | Qualitative study. Semi-structured interviews. | 4 Ways to Wellness provider organisations. | To capture the experiences of patients engaged with Ways to Wellness and to identify the impact of the link worker social prescribing programme on health and well-being. |
| Mtemachani | 2018 | ‘Commissioning social prescribing’ – Practice Management. | Commentary paper. | N/A | To describe the potential of social prescribing in the delivery and reconfiguration of health services to make them more sustainable. |
| Mulimba and Prus | 2016 | ‘How can a care navigator add value to patient experience in accessing health and care services at the right time, right place with the right support and input?’ – NHS England. | Rapid review. Mixed methods. Electronic searches of key organisations and contacting project leads | Qualitative and quantitative papers, project reports, stakeholder websites, press releases | To describe key examples of care navigation in primary care, community health services, the voluntary sector and online |
| Naick | 2018 | ‘Providing telecare for older adults: understanding the care navigators’ experience’ - Quality in Ageing and Older Adults. | Qualitative study. Interviews. | Care navigators from the VCS and telecare installers. | To explore contextual factors that influence care navigators' practices and how they affect the delivery of telecare. |
| National Association of Link Workers | 2019 | ‘Getting to know the link worker workforce  Understanding link workers  knowledge, skills, experiences and support needs’ – National Association of Link Workers. | Report. Quantitative. Questionnaire (with some open-ended questions). | 105 link workers. | To explore the skills, experiences and needs of link workers. |
| Nesta | 2013 | ‘More than medicine: New services for people powered health’ – Nesta, Innovation Unit. | Report. Mixed methods. Case studies and routinely collected data. | Six teams who took part in People Powered Health. | To explain what People Powered looks like and key features associated with its success. |
| NHS England | 2019 | ‘Social prescribing and community-based support Summary guide’ – NHS England. | Guide. | N/A | To guide people and organisations in leading the local implementation of social prescribing. |
| NHS Health Scotland | 2016 | ‘Evaluation of the Links Worker Programme in ‘Deep End’ general practices in Glasgow’ – NHS Health Scotland. | Evaluation. Mixed methods. Data on practice demographics, local community support, focus groups, interviews, documentary analysis. | 7 practices. | To provide an interim report. |
| NHS Wales | 2018 | ‘Social prescribing Torfaen’ – NHS Wales. | Report. Mixed methods. Unclear methods. | 13 GP practices. | To report on social prescribing over a 1 year period. |
| OPM | 2017 | ‘Evaluation of the Care Navigation Service’ – OPM. | Evaluation. Mixed methods. Interviews, focus groups, service user outcome data, economic analysis of hospital usage. | 34 GP practices. | To evaluate the care navigator pilot scheme. |
| Palmer et al. | 2017 | ‘Social Prescribing in Bexley: Pilot Evaluation Report’ – Mind in Bexley. | Evaluation. Mixed methods. Questionnaires, healthcare usage data, interviews, observations. | 9 GP practices. | To evaluate benefits and limitations of the pilot. |
| Penwith Pinoneer Project Board | 2013 | ‘People, Place, Purpose:  Shaping services around people and communities through the Newquay Pathﬁnder’ - Age UK Cornwall & Isles of Scilly. | Evaluation. Quantitative. Questionnaires, healthcare usage data. | Newquay Pathfinder programme (Age UK). | To evaluate a pilot scheme. |
| Pescheny et al. | 2018 | ‘Patient uptake and adherence to social prescribing: a qualitative study’ – BJGP Open. | Qualitative study. Interviews. | 4 GP practices. | To explore the experiences and views of service users, GPs, and care navigators on factors influencing uptake and adherence to social prescribing. |
| Pescheny et al. | 2018 | ‘Facilitators and barriers of implementing and delivering social prescribing services: a systematic review’ – BMC Health Services Research. | Systematic review. Mixed methods. | Social prescribing in the UK. | To identify factors that hinder and facilitate the implementation and delivery of SP services based in general practice. |
| Price | 2018 | ‘Care navigation’ - Herefordshire Community Education Provider Network. | Evaluation. Quantitative. Routinely collected data. | 19 GP practices. | To evaluate the introduction of care navigators into practices. |
| Redbridge CVS | 2018 | ‘Redbridge CVS Report on Social Prescribing Service’ – Redbridge CVS. | Report. Mainly quantitative. Routinely collected data, but did include some comments and case studies. | 9 GP practices. | To update on the progress of a social prescribing service. |
| Resolution Foundation | 2008 | ‘Navigating care: A discussion paper’ – Resolution Foundation. | A position paper. Mixed methods. Literature review, questionnaire, focus groups. | Older people and low earners and organisations supporting them. | To identify what older people and their families need to help with navigating the care system, and how they would like to receive this help. |
| Ridge | 2017 | ‘Leeds North CCG Social Prescribing Evaluation’ – Leeds Intelligence Hub. | Evaluation. Quantitative. Routinely collected data and questionnaires. | One service employed by and delivered in 7 GP practices and another by a consortium. | To evaluate and compare two different operating models. |
| Rodgers et al. | 2016 | ‘Integrated care to address the physical health needs of people with severe mental illness: a rapid review’ – Health Services and Delivery Research Report. | Rapid review. Searched for empirical and descriptive papers, and policy documents, and consulted with an advisory group. | Integrated care within health settings. | To map models of care for addressing the physical needs of people with a mental health problem. |
| RSM | 2017 | ‘Better Local Care Hampshire Multispecialty Community Provider Vanguard: Deep Dive Evaluation Report: Surgery Signposters’ – RSM. | Evaluation. Quantitative. Questionnaires, activity tracking by VCS, clinical outcomes (including A&E and GP usage). | 5 GP practices. | To describe activity, outputs and outcomes from a surgery signposting service. |
| Sandwell and West Birmingham CCG | 2017 | ‘Care Navigation Training Pilot: Attendee Evaluation Report’ – Sandwell and Dudley CCG. | Evaluation (of training). Quantitative. Pre-post questionnaire. | Those on training came from 10 GP practices. | To report on some care navigator training. |
| Serle | 2018 | ‘'Serious consequences' for NHS after negligence ruling over receptionist's advice’ – Health Service Journal. | News article. | One NHS Trust. | Report on high court ruling. |
| Shropshire, Telford and Wrekin Age UK | 2018 | ‘Contract Review, End of year (Q4) Report’ – Age UK. | Report. Case studies. | One care navigation service. | To report on activity for a care navigator service for year end 17/18. |
| Siddiqui et al. | 2017 | ‘Using ‘Active Signposting’ to streamline general practitioner workload in two London-based practices’ – BMJ Open Quality. | Quality improvement project. Mixed methods. Data on telephone consultations and feedback from staff. | 2 GP practices. | To measure the proportion of patients who did not require a GP to deal with their query, who could be actively signposted by reception staff elsewhere, before and after a support intervention. |
| Skivington et al. | 2018 | ‘Delivering a primary care-based social prescribing initiative: a qualitative study of the benefits and challenges’ – British Journal of General Practice. | Qualitative study. Interviews. | Community link workers and VCS organisations. | To investigate issues relevant to implementing a social prescribing programme to improve intersectoral working to achieve public health goals. |
| Smith and Skivington | 2016 | ‘Community Links: Perspectives of community organisations on the Links Worker Programme pilot and on collaborative working with primary health care: A sub-project of the Evaluation of the Links Worker Programme in ‘Deep End’ general practices in Glasgow Final Report’ – University of Glasgow. | Evaluation. Qualitative. Interviews. | 7 GP practices. | To uncover issues relevant to delivering intersectoral work and to gain a better understanding of how those in community organisations view the link worker model of social prescribing. |
| Social Prescribing Network | 2016 | ‘Report of the annual Social Prescribing Network conference’ – University of Westminster. | Conference report. | One conference. | To report on discussions and workshops. |
| Somerset CCG | 2016 | ‘West Somerset Living Better: Pilot Project Initial Report’ – Somerset CCG. | Evaluation. Quantitative. Questionnaires. | 5 GP practices in total. | To inform stakeholders on progress of Living Better Scheme. |
| South et al. | 2008 | ‘Can social prescribing provide the missing link?’ - Primary Health Care Research & Development. | Case study. Mixed methods. Interviews and monitoring data. | 2 GP practices. | To explore the concept of social prescribing and examine its value as a public health initiative embedded within primary care. |
| Stocks-Rankin et al. | 2018 | ‘Unleashing Healthy Communities: Full Report Researching the Bromley by Bow model’ – Bromley by Bow Insights. | Exploratory research. Qualitative. Creative, participatory methods. | Community centre and health partnership (3 GP practices) | To develop a conceptual framework to describe the Bromley-by-Bow model and its relationship to the community so that further measurement of impact is robust and meaningful to local people. |
| Swift | 2017 | ‘People powered primary care: learning from Halton’ - Journal of Integrated Care. | Descriptive paper. | Community Wellbeing Practices delivered in 17 GP practices. | To reflect on the design and delivery of community centred approaches to health and well-being. |
| Tavabie and Simms | 2017 | ‘Career planning for the non-clinical workforce: An opportunity to develop a sustainable workforce in primary care’ – Education for Primary Care. | Qualitative study. Review of job descriptions, workshops with key stakeholders. | Community Education Provider Networks and education providers. | To explore the common competencies and features of non-clinical roles. |
| Tavabie and Tavabie | 2013 | ‘The patient liaison oﬃcer: a new role in UK general practice’ – Quality in Primary Care. | Mixed methods study. Questionnaire, focus groups. | 49 GP practices. | To explore development of a patient liaison officer role in general practice to support delivery of integrated community care. |
| Tavabie and Tavabie | 2015 | ‘The Patient Liaison Officer in UK General Practice Co-ordinating Care for Housebound Patients’ – Quality in Primary Care. | Retrospective cohort study. Quantitative. Healthcare usage data, analysis of care plans, questionnaire. | One GP practice. | To identify ways in which a patient liaison officer might improve care for household patients. |
| Walker | 2019 | ‘Plotting the right path with Care Navigators’ – NHS England Blog. | Blog. | One GP partnership. | To describe the introduction of receptionists signposting and acting as care navigators. |
| We are Snook | 2013 | ‘Enabling health and wellbeing among older people: Capitalising on resources in deprived areas through general practice’ – We are Snook. | Evaluation. Mixed methods. Monitoring data (e.g. on participation), interviews, focus groups, literature review, visual methods. | 3 GP practices. | To develop a system through which general practices in deprived areas can identify older people in need and how them to access resources or to participate in activities. |
| Wellbeing 4 U | 2018 | ‘Social Prescribing Impact Report’ – Wellbeing 4 U. | Report. Mixed methods. Questionnaires and case studies. | 17 GP practices actively referring. | To describe the impact of the service and to make recommendations for future development. |
| White et al. | 2010 | ‘Altogether Better Thematic Evaluation: Community Health Champions and Empowerment’ – Leeds Metropolitan University. | Evaluation. Qualitative. Interviews and participatory workshops. | Altogether Better programmes. | To assess how far the aims for a Health Trainer social prescribing scheme were achieved. |
| Wigfield et al. | 2015 | ‘Age UK’s fit for the future ‘Social Prescribing’ extension project: Evaluation report’ – University of Leeds. | Evaluation. Mixed methods. Questionnaires, interviews. | 3 Age UK services. | To assess the extent to which benefits gained were determined by referral route and to explore the potential impact of social prescribing. |
| Wigfield et al. | 2015 | ‘Age UK’s fit for the future: Project Evaluation Report’ – University of Leeds. | Evaluation. Mixed methods. Questionnaires, monitoring data, interviews, case studies. | 11 Age UK services. | To describe and evaluate the service. |
| Woodall and South | 2005 | ‘The Evaluation of the CHAT Social Prescribing Scheme in Bradford South & West PCT’ – Leeds Metropolitan University. | Evaluation. Qualitative. Interviews. | 2 GP practices. | To examine acceptability, relevance and appropriateness of the scheme, assess its effectiveness in addressing social, emotional or practical needs, identify factor affecting delivery and utilisation. |
| Woodall et al. | 2018 | ‘Understanding the effectiveness and mechanisms of a social prescribing service: a mixed method analysis’ – BMC Health Services Research. | Mixed methods study. Questionnaire, interviews, focus group. | A social prescribing service that deploys well-being coordinators. | To understand outcomes of the service and processes supporting its delivery. |
| York CVS | 2019 | ‘The impact of social prescribing in York’ – York CVS. | Social return on investment evaluation. Mixed methods. Questionnaires, data on GP usage, number of new volunteers, number of people engaging in physical activity, interviews. | One primary care medical group | To explore how the service created social value and brought life-changing outcomes to clients. |
