# Supplementary material for: Supporting social prescribing in primary care by linking people to local assets: a realist review
Source: BMC Med. 2020 Mar 13;18:49. doi: 10.1186/s12916-020-1510-7 (PMC7068902; doi:10.1186/s12916-020-1510-7)
Supplement: Supplementary file 5 — Additional file 5. Final, refined realist analysis, with data extracts. [file 12916_2020_1510_MOESM5_ESM.docx]

| **Additional file 5: Refined realist analysis underpinned by a social capital and patient activation lens (with supporting data)**  ***LW = link worker, HCP = healthcare professional, SP = social prescribing, VCS = voluntary/community sector***  ***Structural/personal antecedents to social capital development (required for SP connector roles to have a good chance of working by providing the foundations for trust, credibility and connections to be forged)***  1. There is a vibrant local VCS, offering a range of activities, which the LW has a good knowledge of and patients can access (C), meaning LWs have choice and flexibility (M) so can address diverse patient needs (O).  *“…knowledge of community organisations and the links to the third sector as well as the knowledge of its ‘modus operandi’ was an important skill SPCs could deploy in assisting GPs.” (Bertotti et al., 2018: 242)*  *“…capacity for some popular services can…be outstripped by sharp increases in demand. This has also led to a reduction in the availability of re-referrals for some patients who may need that choice.” (Envoy Partnership, 2018: 34)*  *“Success of meeting patient needs depends on wide range of good quality, flexible community based services.” (Mulimba and Prus, 2016: 11)*  *“There is a need to continue to fund Voluntary and Community Sector services so that they can be signposted to. Both organisations reported a reduction in the capacity of the Sector to respond to needs and that waiting lists are longer. Community Navigation has seen a reduction in the options for support available to people.” (Community Works, 2017: 26)*  *“‘…the only places where you'd struggle to get social prescribing off the ground, is somewhere that’s got a weak voluntary/community sector or they haven’t got very many voluntary organisations around cos it does rely totally on what's available in an area.’” (White et al., 2010: 34)*  *“Without a range of options to offer to service users, the social prescribing service may not be able to address the needs of all individuals.” (Woodall et al., 2018: 10)*  *“The availability of support from voluntary and community sources varied…meaning that Navigators operating in areas with highly developed community services were able to support clients to access support more easily than areas that were lacking.” (Barber, 2017: 48)*  *“These kinds of referrals work well enough for physically mobile populations, but less well, or not at all, for those largely confined to home for mental or physical health reasons, or for reasons of geography or financial barriers.” (Abel et al., 2018: 2)*  *“…transport has been cited by clients from across the area as a problem. The availability of public transport…may be very different to other large metropolitan and urban areas where pilots have taken place.” (Baines, 2015: 34)*  2. Influential figures are consulted when developing the service (C); they understand how it might benefit patients (M), so are supportive of it in discussion with others who are key to its realisation (O).  *“This way of working is still deeply counter cultural (even when a formal system might claim*  *to have embraced it). Senior leadership need to provide genuine top cover and demonstrate ‘courageous patience’ and visible support.” (McGregor et al., 2017: 71)*  *“The partners at our practice have been on board with the programme from the beginning, as they can see the benefits of increasing the skills of some of our existing staff to make a positive impact on patient experience and free up GP time for those patients who really need their specialist clinical support.” (Walker, 2019: NS)*  *“Hearing the message from trusted peers or those with power and influence has encouraged GP practices to engage. ‘We had two very engaged GPs, our CCG clinical leads, who were very supportive. They worked alongside me to get the right sort of comms out to the practices with the right messages.’” (Fullwood, 2018: 24)*  *“The GP Champion was key in translating the ‘blue-sky’ ideas in the pilot strategy into practical solutions for the project plan and pathway. He had also been key to raising the profile of the programme and championing the programme in his own practice.” (Healthy Dialogues Ltd, 2018: 23)*  3. By consulting with key stakeholders, a SP connector role is designed in line with existing practice systems (C), so is regarded by HCPs as easy to use (M) and as a valuable addition to what they can offer (M), meaning they welcome the service and are prepared to refer patients to it (O).  *“Engaging with GPs and practice managers during co-design…This has ensured that a cohort of GP practices are engaged enough to refer older people when the service ‘goes live’.” (Fullwood, 2018: 24)*  *“The referral system was generally regarded as effective, and the consultation at the outset with the health care professionals was an important factor in this. ‘I think what helped massively was that it actually made the referral easy. Before, we didn’t actually know how to do it…Now it’s fantastic, there is actually one form with all the available services, and you literally select and tick the right one and it’s very simple and straightforward.’” (ERS Research and Consultancy, 2013: 38)*  *“All seven Intervention Practices developed bespoke methods for referring patients to the CLP. Some used the EMIS practice patient record system, others handwritten notes, one used on-the-spot referrals (whereby the GP introduces and refers the patient to the CLP personally), others adapted existing practice templates. Some combined these methods. The main point is that the referral systems were developed over time by practice staff themselves, and thus were able to fit into practice routines.” (Mercer et al., 2017: 46)*  *“We met with staff at all 17 GP practices prior to launching the CWP model to seek their ‘buy in’. These preliminary meetings provided useful insights into how health professionals believed the model could support patient’s needs.” (Swift, 2017: 165)*  4. Accessible, transparent information about the service is available for HCPs (C). Their understanding of the LW role is strong (M), so they are clear about what to expect and recall it when faced with a situation where a LW could help (O).  *“Ensure that GPs are fully aware of the role and its use within practices and the wider community, such that they attain the confidence in utilising the PCN role at the Practice.” (Deloitte, 2015b: 26)*  *“Advice workers stressed the importance of promoting the service to practices, including offering initial training for staff about appropriate referral.” (Greasley and Small, 2002: 3)*  *“…building trusting relationships and demonstrating credibility – both been critical to securing the engagement of GPs and other health and care professionals…Send practices concise and compelling summaries of the support given to all their PICP patients – not just individuals – and the goals achieved overall. This will strengthen GPs’ understanding of the scale of the value offered and is likely to boost their engagement.” (Fullwood, 2018: 24)*  *“Feedback from project staff and GP/practice staff indicates that a lack of clarity over the type of patient to be referred to the project has been one factor in the lower than expected uptake to date.” (Baines, 2015: 22)*  *“’The terrible thing is that I referred five but I should have referred about 15 times that. Although I am very enthusiastic about it, it is hard to keep in front of your mind, and that’s the challenge!’” (General Practitioner)” (Bertotti et al., 2015: 15)*  *“With competing demands for time and resources, one GP suggested that Living Well was “on the margins” of his radar.” (Leyshon et al., 2015: 8)*  5. LWs work in a supportive environment (e.g. number of referrals remains manageable, peer assistance, supervision from managers, training, job security, welcomed by practices) (C), enabling them to feel confident in what they do (M) so they can function effectively within the role (O).  *“Social Prescribers often had patients on their caseload with complex health and social care problems and complex navigation for onward referral, for which they had a degree of clinical and emotional responsibility but very little support in terms of clinical supervision. Social Prescribers also felt that they were often burdened by administrative tasks which may detract from the focus of their role.” (Ferguson and Hogarth, 2018: 41)*  *“The monthly national learning forum is a reflective community of practice…It has provided a source of inspiration and a welcome opportunity to share knowledge, challenges and effective practice. Importantly, the forum has sought to create a ‘positive error culture’, where participants are encouraged to talk openly and honestly about what isn’t working, mistakes and uncertainty.” (Fullwood, 2018: 38)*  *“We suggest creating posts for more experienced CLWs within the team, who can play a supervision and management role, as well as having a caseload. This should enhance the quality of the service and help retain talented professionals within the team.” (Innovation Unit, 2016: 34)*  *“Social prescribing link workers need regular access to ‘clinical supervision’ to support them in their connecting roles. Link workers often see people in crisis and vulnerable situations. To be effective, the issues people present, including domestic violence, sexual abuse, family dynamics, self-harm and suicidal thoughts, need to be heard in a safe supervision space. Link workers need dedicated time to offload and to have clear safeguarding procedures to deal with situations appropriately.” (NHS England, 2019: 13)*  *“…staff who are employed via temporary contracts to support SP pilots (e.g. navigators or project managers), may seek alternative more stable employment, as the future or prospect of their roles might be unclear (Pescheny et al., 2018b: 9)*  *“One advice worker described what constituted a good and a bad GP practice: 'Good: took time to get to know me personally, and my role; they ring me to check if the referral is appropriate. Bad: they put me in a room; reluctantly make appointments; There is no interaction.” (Greasley and Small, 2002: 3)*  6. Patients are referred at a stage in their life when able to make best use of support provided by a LW and are seen in a timely manner (C); they are receptive to ideas (M) and have the energy (M) to contemplate trying something new (O).  *“…often clients are suffering from a lack of confidence and or anxiety related conditions and do not feel capable of engaging with the scheme. Another factor may be that some clients are leading chaotic lives and do not see the potential benefit that may accrue.” (Baines, 2015: 33)*  *“Hard to make inroads when complex cases – sometimes have pressing needs with money, family issues – have to address before can move onto social issues.” (Green and Ellerby, 2017: N/S)*  *“‘It (social prescribing) gave me the motivation to think I might be ready to go back to work. It helped me deal with my depression, prepared me to go back to work and made me feel useful.’” (Patient) (Bertotti et al., 2015: 7)*  *“Not all service users access the services that they are referred to, for a number of reasons, for example not being ready to seek further support or competing life issues.” (Ferguson and Hogarth, 2018: 43)*  *“Timing of advice is crucial – for many people, a life event is the only way they are prompted to action. Unless their situation changes (particularly for the worse) – they will lack motivation to act upon advice.” (Resolution Foundation, 2008: 34)*  7. The patient is given clear information about the service by a trusted HCP (C); hence, they believe it is a worthwhile avenue to pursue (M) so are willing to see a LW (O).  *“Stakeholders involved in the development of social prescribing argued that GPs are in a powerful position to advocate a new approach to their patients as they have a consolidated reputation.” (Bertotti et al., 2018: 237)*  *“Informing patients of the service was a crucial element of the referral process. Most staff reported making a conscious effort to ensure that patients had a full understanding of the scheme before progressing with the referral.” (Woodall and South, 2005: 13)*  *“‘People come to the GP and expect to find all the help they need. It is great to be able to offer this kind of support (CLW) within the practice and people trust it because they associate it with their GP.’” (Innovation Unit, 2016: 40)*  *“Having CLPs situated within GP practices was viewed positively, as it allowed access to primary care health professionals but also because of the standing that GP practices have within communities owing to their longevity and to the feeling that patients hold a certain level of trust in their GP practices...” (Smith and Skivington, 2016: 4)*  *“…older people have proved more receptive to the invitation to take part in the programme if their GP, as someone they trust, made the introduction verbally, rather than just sending a letter.” (Fullwood, 2018: 32)*  *“For GPs, there was an initial challenge in getting patients to agree to engage with the Social Prescribing Service. This included ensuring that patients understood the purpose of the service and then accepted it as a viable complementary service…It was argued that all primary care services (i.e. not just the GP) needed to support and encourage patients to engage with Social Prescribing...” (Dayson and Moss, 2017: 3)*  8. The LW spends time in primary care and in VCS settings (C), so comes to understand the culture and language in both (M), meaning they are regarded as a trusted, credible boundary spanner (M), who can facilitate joint working between professionals from different backgrounds (O).  *“There were also culture differences that were subtle but important for appreciating the complexity of the pathway: GPs referred to ‘patients’, SPCs to ‘clients’, and some voluntary organisations to ‘people.’” (Bertotti et al., 2015: 14)*  *“Navigators…act as a bridge between community services, groups, activities and GP surgeries, creating better two way communication and relationships based on increased awareness and understanding.” (Mulimba and Prus, 2016: 9)*  *“Where participants felt that they had a “route in” (CO23) to GP practices via the CLP, they were more positive about being able to collaborate with primary care. They felt that for this to work the link had to carry “weight, gravitas, authority and credibility with a GP practice” (CO16).” (Smith and Skivington, 2016: 16)*  *“…CLPs played an important and specific role in sharing knowledge of how primary care works with staff in COs. This could be critical to really cementing relationships: ‘So the warmth is coming from the relationships that the community links practitioner is able to develop because she’s there in a position to actually understand the dynamics of a surgery, which are really full-on. I mean, it’s high pressure...’” (Mercer et al., 2017: 51)*  *“Link worker phoned the community organisations and arranged to visit them or for them to come into the practice.” (We Are Snook, 2013: 24)*  *“One of the respondents pointed out that the community sector has traditionally found it difficult to establish links with GP practices directly. Social prescribing provides a vehicle for the council and the CCG to encourage GP practices to refer patients to the voluntary sector.” (Bertotti et al., 2017: 27)*  *“The Social Prescribing team attend the practice team meetings and Patient Participation Group meetings. They have built strong working relationships with local voluntary sector organisations that provide social and practical support. The team have met with each of the organisations to review their referral process to the VSOs, to ensure appropriate signposting to the most appropriate services for the patients and provide feedback as necessary.” (Edwards, 2018: NS)*  *“Navigators felt that health and social care professionals didn’t have the time to understand the kinds of community support that may available for clients. This meant that they viewed themselves as the key link between statutory services and the voluntary/community sector.” (Barber, 2017: 39)*  ***Social cohesion (relates to the establishment of trust, credibility and connections)***  9. When LWs interact (e.g. through feedback, presenting at meetings, visiting services) with VCS providers and HCPs they can demonstrate their knowledge and ability to help patients (C), engendering trust in their skills and competency (M). Consequently, HCPs are willing to refer to and the VCS will take referrals from LWs (O).  *“It was noted that having a link worker based in surgeries, and using the same computer system allowed for enhanced communication between healthcare professionals within the practice and the link worker.” (Social Prescribing Network, 2016: 24)*  *“The number of individual GPs and GP practices that have referred to the service has increased throughout the 22 months of operation. Stakeholders attribute this to a range of factors including ongoing awareness raising/engagement activity by care navigators and the CCG, closer working relationships with clinicians ‘on the ground’, and clinicians observing the benefits and outcomes of the service.” (OPM, 2017: 14)*  *“‘The advisors are personal, professional and get to know the client…They give me a lot of clear information which is helping me to understand their role…they have to be efficient because they are seeing a lot of patients...’ (VCS Provider)” (Dayson et al., 2013: 16)*  *“…staff felt comfortable with referring patients as they had high levels of trust and confidence in the CHAT worker’s enthusiasm and ability to find appropriate voluntary sector services.” (Woodall and South, 2005: 15)*  *“GPs who engaged with the service more fully tended to have a higher level of trust in their Navigator than those who did not, which in turn affected the number of referrals made to the Navigator.” (Farenden et al., 2015: 43)*  *“…stakeholders noted that the PICs’ grasp of ‘what’s available in the community’ extended beyond ‘what’s out there on paper or on the internet’ to having a real understanding of the nuances of different clubs, groups and services…‘They have a big knowledge base of what is happening locally.’” (Fullwood, 2018: 18)*  10. The boundary spanning work of the LW (C) engenders a greater appreciation by HCPs of the VCS (M) and trust in these services (M) so they are willing to treat VCS providers as equal partners in helping to resolve patients’ non-medical needs (O).  *“GPs were already aware that there was ʻlots going on in Dundeeʼ but faced considerable barriers to acting on that knowledge and signposting patients. These included the diﬃculty of gaining and maintaining up to date information and the time involved, lack of knowledge and conﬁdence that speciﬁc services/groups were competent, reliable and of good quality, and not knowing enough about a service or activity to be conﬁdent that it would meet a particular patientʼs needs.” (Friedli et al., 2012: 26)*  *“For one GP, the presence of the CLP had increased confidence in the organisations:*  *‘… what I'm finding more and more is I can say to patients “Have a think about this organisation. We've got a few people who've found it helpful” and I can do that with confidence because I know [the CLP] has maybe been with one person [to the organisation] then sent another, you know?’ GP 5, Partially Integrated Practice, in-depth interview (Mercer et al., 2017: 33)*  *“Those with an interest in the voluntary and community sector saw social prescribing as potentially beneficial to this Third Sector, enhancing tenuous links between health services and groups, reducing the mistrust between them…” (Brandling and House, 2007: 13)*  *“…raising awareness among statutory agencies of the contribution the voluntary and community sector can make to health and social care delivery.” (Wigfield et al., 2015a: 6)*  *“For the Community Navigation service to continue working effectively in the long-term, it is vital that partnerships between the Health and Community and Voluntary sectors continue to develop according to shared goals and an understanding of the unique strengths each partner brings to the service.” (Farenden et al., 2015: 38)*  *“In many areas, social services, community services and general practice have grown apart since the inception of the NHS, whereas the integration of services requires that they work together to develop understanding and unity of eﬀort to achieve ‘mutual beneﬁt’ and improved care for patients.” (Tavabie and Tavabie, 2013: 304)*  *“There can also be an educational element in terms of increasing health professionals’ awareness and understanding of voluntary organisations.” (South et al., 2008: 316)*  11. Patients see the LW in an atmosphere that is conducive to discussing their needs (e.g. they are not rushed, are asked questions in a sensitive manner, are seen in their own home) (C). They feel comfortable (M), valued (M) and listened to (M), so are prepared to talk openly (O).  *“Advisors talked about the significance of seeing people in their own home…in terms of building rapport and trust…Advisors felt that clients were more likely to 'open-up' to them and discuss problems and concerns if they were in their home environment.” (Dayson and Bennett, 2016: 3)*  *“Trusting relationships are built over several home visits, enabling the older person to express their desires and needs freely…Unprompted, clients across all sites consistently described the guided conversation as being ‘like chatting to your friends and family’, and spoke about feeling valued, listened to (rather than ‘talked at’) and in control.” (Fullwood, 2018: 17)*  *“Patients’ interaction with SPCs was key to their progression and experience…Patients spoke positively of being given the time and space to discuss their needs, feeling heard…” (Bertotti et al., 2015: 6)*  *“…the CHAT worker is able to spend longer with a patient than primary care staff are often able to, offering up to three forty-minute appointments. This provides the patients with the opportunity to discuss any issues that they feel are affecting their health and the possibility of exploring a variety of solutions.” (Woodall and South, 2005: 2)*  *“…some clients found it diﬃcult to talk about their problems…link workers were able to help patients to open up and the clients appreciated this. ‘It was scary to start with having to go and open up to somebody a bit…It takes a couple of sessions to really feel trust.’” (Friedli et al.,2012: 21-22)*  *“…the SPC gave the time to explore their situation; patients had up to one hour to talk in their initial meetings. Participants stated the SPC uses that time to listen without rushing, jumping to solutions or making judgements.” (Healthy Dialogues Ltd, 2018: 41)*  ***Social capital (cognitive, psychological or instrumental resources acquired from developing trust and connections) and its consequences***  12. During conversations with the LW, the patient is permitted to consider their own needs and well-being goals (C). They no longer feel alone (M) and believe life could have meaning (M), resulting in a more positive outlook (O).  *“The Link Workers’ person-centred and non-judgemental approach facilitated…feelings of control and a readiness to reflect on current circumstances and implement positive changes.” (Moffatt et al., 2017: 6)*  *“…one service user reported: ‘She was just absolutely wonderful…she was just right…I told her what had happened and that seemed to get it out of my head a bit. All these years it’s just been in my head.’ (service user)” (Bertotti et al., 2018: 238)*  *“‘I always make it very clear it’s about the goals they want to set for themselves. This is often the first time they’ve had to generate ideas for themselves, used to being told what to do by the GP. Tell me what to do! Well what do you want to do? That’s quite a big thing, I think, for some people.’” (Bertotti et al., 2015: 19)*  *“The client said ‘Having the right support helped me make the right decisions and feel confident to take the right steps. I feel like my life is worthwhile now.’” (Hogarth and Ferguson, 2018: 30)*  *“Patients also appreciated the way SPHTs helped them to think through their situation and come up with a way forward.” (White et al., 2010: 15)*  13. LWs have time, skills and knowledge to act as catalysts for change (C); they can nudge people and empower them to shift their mindset (M), meaning they are prepared to try new things (O).  *“‘It felt like I had someone beside me supporting me through it.’ (Service user)” (Impetus, 2017b: 3)*  *“Before they had the PICs’ support, many clients involved in the research across all sites said that they had been unaware of the help available and/or had not known how to access it…Many clients mentioned that the PICs had been able to arrange access to practical and medical assistance especially much more quickly than other services they had experienced. Clients described being surprised and pleased at how fast the PICs had ‘got things moving’...” (Fullwood, 2018: 13)*  *“Until March 2015 the Navigators were able to provide the signposting information but not to contact the service provider and make the arrangements for the client to attend. It has been recognised that this is a weakness as many clients lack confidence and Navigators now do make arrangements for clients to attend activities.” (Baines, 2015: 15)*  *“Most interviewees described the wealth of information that the SPC has to hand and provides to them. They appreciate the knowledge and connections the SPC has with the services within the community. ‘He got me in touch with places I didn’t even think about, I didn’t know that was there, yet it was across the road.’” (Healthy Dialogues, 2018: 42)*  *“’Not having accessible transport to get to activities is an issue for the majority of patients. There will always be a demand for specialist transport services but using community transport has met some of this demand.’ (VCS Provider)” (Dayson et al., 2013: 15)*  *“‘…when I ﬁrst began I was quite…I rarely went out, I rarely did anything. It kind of gave me that boost to go and do things with the kids and I was always kind of worried about money. So she put me on to places that didn’t cost too much or were free sort of activities...’” (Friedli et al., 2012: 22)*  14. Meeting with the LW, the patient is supported and encouraged to develop social connections (C), through which their confidence (M) and sense of resilience increase (M), so they feel more able to cope with life (O).  *“The Navigator’s relationship with patients is facilitative, empowering and short-term. They encourage and enable people to take up groups, services or activities and do not create dependence on the Navigation service itself.” (Farenden et al., 2015: 10)*  *“The motivation and support offered by SPCs creates the basis for behaviour change such as greater willingness to participate in chosen community activities.” (Bertotti et al., 2018: 240)*  *“Building self-confidence, self-reliance and independence was another facet of the Link Workers’ approach, managed through ongoing support and persistence in finding the right motivational tools for the individual, while conveying the need for personal responsibility…'After [partner] passed away I was, not a recluse, but I just didn’t want to talk to anybody. But since I’ve been coming to see [the Link Worker] I’ve broadened my horizons and I get out … I’ve got a lot more confidence.'” (Moffatt et al., 2017: 6-7)*  *“Another participant saw a direct relationship between increased social engagement and her increased confidence: ‘It [the chair-based exercise group] gets you out of the house. You have to do that because you can feel a bit lost at times. You feel more confident if I you are part of something.’” (Wigfield et al., 2015a: 48)*  15. Engaging in social activities with support from the LW, and meeting new people as a consequence (C), means that patients are distracted from their own situation (M) and less focused on their difficulties (M) as they develop an alternative, more positive mindset (O).  *“Having the opportunity to attend support groups in the local community, facilitated through the social prescribing service, enabled individuals to gain more of a balanced perspective by being able to share experiences with others going through similar difficulties. This resulted in some individuals feeling much more hopeful about their own lives: ‘Since I went to that group I could see what other people are actually having difficulty in life with, and you do not assess yourself the same. It actually made me realise that life is not all about yourself. You find here that everybody has got different problems. You find that yours is not even as serious as the other person that you are talking to.’” (Woodall et al., 2018: 5)*  *“I’ve met a lot of new people … and I realised that people have the same type of you know, same type of lifestyles, maybe lost their job or their partner but they went out there. We have a laugh and I feel better in myself.” (Wigfield et al., 2015a)*  *“‘We don’t see her anymore, the GP keeps saying to me, ‘I haven’t seen such and such for a long time’ and I keep saying, ‘Because she’s too busy doing things for me to come and see you!’ There are so many stories like that that we can tell, through people becoming Practice Health Champions but also then people going to the groups that the champions have set up.’” (McGregor et al., 2015: 6)*  16. A more positive outlook (C) makes patients relaxed and easier to be around (M), allowing them to forge better relationships with family and friends (O).  *“The Social Prescriber accompanied her to her first gambling counselling support session, as she was reluctant to attend. She is now attending sessions with her husband and feels their relationship has improved because they are dealing with the problem together.” (Ferguson and Hogarth, 2018: 36)*  *“Engagement with Alzheimer’s Society has reduced the conflict in their relationship and both are much calmer…The daughter said ‘this is the first time they’ve been able to do this in five months. I feel it is because of how positive and calm Mum was after the singing sessions. I am so grateful for the support they’ve had.’” (Palmer et al., 2017: 20)*  *“Over time, the patient has reported significant reductions in both alcohol and smoking and is no longer taking sleeping tablets and her relationship with her son has greatly improved.” (Wellbeing 4 U, 2018: 18)*  *“…family members noted positive changes made within the family unit as a result of their involvement in the social prescribing service. One individual described how her family members were less anxious regarding her wellbeing and state of mind, ultimately having an impact on their own wellbeing: ‘I have two teenage daughters. I think it was very tough for them. They didn’t understand other than the fact they could see mum was really upset and struggling with things. And they did help a great deal and they are still helping me although not as much as they did [Laughs].’”*  17. Developing new connections and a more optimistic mindset (C) means that patients can see a range of solutions to their difficulties (M) and no longer regard their GP as a first port of call (O).  *“Ten of the patient participants thought that the PSS would, or had reduced their GP attendance. Several reasons for this were evident in the data. Firstly, in cases where patients were gaining assistance from alternative sources (i.e. from the PSS itself or community-based services), it was felt that this negated any need to visit the GP.” (Faulkner, 2004: 44)*  *“One client in particular felt that the Social Prescribing Service had made her almost forget her GP as she felt so well supported through the Social Prescribing Advisor, but also the services onto which they had been referred. ‘I don't think I went to the GP a lot, no, because quite honestly, with the lady that was coming, you nearly forget the GPs there to go to. You do, I never give it a thought, because they were coming.’” (Dayson and Bennett, 2016: 21)*  *“‘We haven’t seen as much of some of our patients since the service, where previously we were their point of contact for non-medical things that we don’t support…they have had someone else to go to or someone to talk to about things, or that the PICs have put in place support to help with their social issues has driven that difference.’” (Fullwood, 2018: 15)*  *“Evaluation of the service across two GP practices examined the impact of service, demonstrating a 50% reduction in GP attendances six months after accessing care navigation/health coaching pathways…” (Health Education England, 2016: 11)*  *“Offering a solution outside of the health realm may break any cycle of patient passivity and dependence as well as removing elements of health professional paternalism.” (Brandling and House, 2007: 21)*  *“…if we look at the potential savings from reduced dependence on GP services we can see that patients who received social prescribing were visiting GP practices 1.2 fewer times over six months.” (Kimberlee, 2016: 35)*  *“There was a significant reduction in the recorded number of patient appointments (telephone and face-to-face) with GPs and other practice staff (z = 2.90, P = 0.003)…” (Grayer et al., 2008: 4)*  *“There was a statistically significant reduction in the average number of GP appointments three months after accessing Ways to Wellbeing. The total number of appointments held by Ways to Wellbeing clients prior to accessing the service was 210, and the total after was 143. This is a 32% reduction in overall appointments.” (York CVS, 2019: 18)*  *“A second client felt that she was attending her GP less, because she was less anxious: ‘I probably go [to the GP] less now, because I'm not sat here worrying about things as much, I'm more active, and getting out there and doing things.’” (Dayson and Bennett, 2016: 21)*  18. In meetings with the LW the patient can offload their troubles (C). They feel cared for (M) and unburdened (M), so enjoy talking to this person (O).  *“…talking to an Age UK support worker…improved her mood: ‘When [the support worker] from Age UK calls, she asks me how I am, what sort of exercise I get, everything in general really…It helps me to talk about things.’” (Wigfield et al., 2015a: 49)*  *“The opportunity for patients to be listened to in-depth for a period of time was a new experience, and they valued the opportunity to share their problems in a friendly and non-confrontational environment: ‘I had a chat with xx…he was really really nice…it’s often easier to talk to a stranger than people you know…’” (Woodall and South, 2005: 19-20)*  *“Mrs. T: ‘You have helped me so much since my husband died. After being married 56 years I did not know which way to turn. You have done all sorts and have been there on the end of the telephone when I have needed someone to listen to me’.” (Andrew, 2016: 19)*  *“…patients were comforted by the thought that they could talk to the service in conﬁdence, that the service would listen to their problems, and that they would receive well-informed advice.” (Faulkner, 2004: 44)*  *“They valued the officers’ personal qualities and described them as empathetic, non-judgmental, empowering, effective communicators, good listeners, competent, supportive and helpful. They suggested that this enabled them to talk about difficulties and sensitive issues.” (Aitken et al., 2017: 9)*  *“Interestingly, those patients who were unsuccessful in locating or attending voluntary sector services still felt positive benefits from having time to talk through issues with the CHAT worker. Being listened to, respected and understood were important for patients and improved their outlook and esteem.” (Woodall and South, 2005: 27)*  19. Patients who have significant physical and/or psychosocial difficulties (C) may experience emotional relief from meeting with a LW (M) but require ongoing professional support due to the nature of their situation (O).  *“…for a minority of clients the intervention by the Navigator service had not led to any noticeable improvement in some of the core health and wellbeing problems…These clients had long term physical health and/or severe mental health problems that they…were not always amenable to support available from the voluntary or community sector. Nevertheless, these clients still highly valued the Community Navigator service and felt it was an important part of their life…improving their emotional wellbeing.” (Barber, 2017: 35)*  *“…possible explanations as to why we did not identify overall reductions in service use. Foremost, the large majority of people who accessed the care navigator service had a disability, and many have multiple disabilities and complex needs. A large number were also frail and housebound. Additionally, the service is targeted at elderly people, many of whom were aged 85 years or older. As service users continue age, health status and independence may deteriorate, requiring greater use of services.” (OPM, 2017: 44)*  *“…severity of the challenges posed by those patients who were referred meant duration of engagement with the service was often greater than initially planned.” (Kimberlee, 2016: 27)*  20. The patient feels a connection with the LW (C), which means they feel safe to open up about previously undisclosed issues (M) that have to be referred back to a HCP (O).  *“‘They're easy to talk tae. I can open up. I feel as if I can open up to [CLP name]. I have opened up to [CLP name], do you know what I mean?’” (Mercer et al., 2017: 46)*  *“…the social prescribing coordinator has been able to identify additional medical or safeguarding issues as a result of the social prescribing referral and as such, has been able to raise and act on these issues with primary care professionals.” (Palmer et al., 2017: 17)*  *“A recent review of 38 schemes that integrated health and social care funds challenged the assumption that integrated funding leads to better health outcomes and lower costs. Rather, improved integrated care tends to uncover unmet needs, with total care costs likely to rise.” (Rodgers et al., 2016: 18)*  *“In a linear regression analysis (with annual consultation rate as the outcome), after adjusting for age, gender, ethnicity, co-morbidities and general practice, the annual consultation rate in cases was confirmed to be higher than in controls (beta coeff= 2.79, p=0.013).” (Carnes et al., 2015: 16)*  *“A further, unintended effect of the pilot has been to make referrals to statutory sector services…Although it cannot be said for certain that these referrals would not have occurred eventually through other means, in many cases it has ensured that the referral happened sooner rather than later.” (Dayson et al., 2013: 10)*  *“…social prescribers have been able to pick up on medical issues that the patient may have been too embarrassed to share with a GP. This includes issues like alcohol dependency. In one district they claim that 40% of their referred patients discussed their problem of alcohol dependency with their co-ordinator.” (Kimberlee, 2016: 31)*  *“The ability of the social prescribing coordinator to identify additional needs, and raise these with relevant healthcare and support professionals provides a valuable service…It is important to note that identification of these additional requirements may actually result in increased costs to health and social care services immediately after a social prescribing referral…” (Palmer et al., 2017: 17)*  *“4% (n=16) of patients were referred back the GP with complex needs that required specialist support or care co-ordination.” (Farenden et al., 2015: 22)*  *“In some instances, responding to unmet need will lead to an increased use of statutory healthcare services resources. Indeed, the analysis of the support clients receive to achieve their goals highlights that while referrals to social care account for less than one percent of all referrals to other services, a minimum of 286 clients have been referred to the NHS, particularly primary care, through their involvement in the programme.” (Fullwood, 2018: 15)*  21. When sessions with a LW end for 'dependent' patients (C), they feel abandoned (M) or angry (M) and might return to seeing their GP for emotional solace (O).  *“While CLWs invest in building trusting relationships with people and making them feel like someone is there to help them, they also need to carefully manage the risk of creating a relationship of dependency with the people they support, particularly those with more complex needs.” (Innovation Unit, 2016: 36)*  *“…a number of clients found it hard to bring about sustained behaviour change, and continued to rely on the Navigator. In some cases clients felt that the support from their Navigator needed to continue indefinitely.” (Barber, 2017: 34)*  *“…there is no discharge guideline or policy that the programme follows, rather the patient will see the SPC until they no longer need the service or they stop attending. This has not posed any issues for the programme in this one year pilot. However, a well defined set of guidelines on discharging patients will empower the SPC to support clients to transition away from the service once they complete the intervention.” (Healthy Dialogues Ltd, 2018: 27)*  *“Some patients are highly complex and they can take the view that the coordinator is a ‘support worker’, taking little responsibility for their participation but demanding high levels of input.” (Wellbeing 4 U, 2018: 20)*  *“On the flip side of the relational and motivational nature of interactions, there is the risk that the strong connection between the client and CLW may create a sense of dependency. Particularly in the absence of a clear process and expectations around clients’ exit from the service, both clients and CLWs may find it difficult to decide when it is time to end an intervention.” (Innovation Unit, 2016: 39)*  *“…some people were angry they could not be helped by the care navigators more. It was reported these individuals asked for help beyond the abilities of the care navigators. In particular, assuming care navigators would be around in the long-term and work in a life-coach role, rather than supporting self-management to occur.” (Darnton et al., 2018b: 17)* |
| --- |

| ***Contribution of patient activation***  22. Seeing a LW and co-producing a personalised action plan with this person (C) allows the patient to develop agency in a safe space (M), which encourages them to start taking ownership for their well-being (O).  *“Navigators work to develop ‘I’ statements, personalised care plans which build upon a person’s goals, desires and needs.” (Health Education England, 2016: 39)*  *“The person is supported to tell their whole story, identify and prioritise key issues and challenges they may face and then support the person to make a decision on how to move forward to resolve the situation or move towards a behaviour change.” (Wellbeing 4 U, 2018: 8)*  *“A large part of the reason for the popularity of the service with patients is the supportive, problem solving model social prescribing health trainers use which puts patients in control of how they address the issues they want to tackle.” (White et al., 2010: 4)*  *“Link workers may also suggest appropriate resources and support for the patient to access…The aim is to provide the patient with a ‘voice’ in this process.” (Social Prescribing Network, 2016: 9)*  *“The use of guided conversation and facilitated referral methods put patients at the heart of their navigation journey. This collaborative relationship provides an empowering space for patients to explore options and make decisions about the support they access, encouraging informed choice and promoting patient self management.” (Farenden et al., 2015: 33)*  *“Service users appreciated not feeling under pressure to agree to activities suggested by navigators, if they didn’t like them. Empowering and actively involving service users in decisions on their onward referrals facilitated feelings of control.” (Pescheny et al., 2018: 8)*  *“Providing a guiding hand, rather than taking the lead, promotes autonomy and the motivation to succeed.” (York CVS, 2019: 24)*  *“This collaborative process enables individuals to feel confident enough to take responsibility for their own wellbeing, as well as allowing them to recognise that they themselves are perfectly able to make changes in order to improve their health…” (Bunyan et al., 2017: 17)*  23. Attending groups and activities discussed with the LW (C) prompts patients to feel socially connected and supported (M), increasing their motivation and self-confidence to manage their own well-being (O).  *“…the link worker (job title variously named) met with the patient to discuss their needs and directed them to appropriate community/voluntary sector sources of support in their locality.” (Bikerdike et al., 2017: 3)*  *“After being referred to Social Prescribing Mr B started going to a gym once a week, and participated in activities at the community centre, including creative writing, on several other days. Since receiving support through Social* *Prescribing Mr B had become more independent and positive.” (Dayson and Bashir, 2014: 26)*  *“Some patients said that after seeing a CLP and visiting a community resource, they felt better able to find help themselves and self-manage problems.” (Mercer et al., 2017: 3)*  *“‘Reduction in social isolation…increased social interaction, which has an effect on quality of health…more likely to take care of oneself, and be alert…People have made friendships within the group and do telephone each other.’” (Dayson et al., 2013: 21)*  *“…it was thought that the scheme increased the awareness of patients of the VCS services available in the borough, supporting them to manage their own health through better use of wider community assets.” (Ferguson and Hogarth, 2018: 37)*  *“‘I am able to access a range of community support to help me maintain my resilience and wellbeing.’” (Community Works, 2017: 23)*  *“The largest change (67% to 78%) was in patients’ sense of self-worth and motivation to take care of their own health and medication, and feeling more valued by the health care system.” (Envoy Partnership, 2018: 23)*  *“People report improvements in all of the questions relating to their confidence in the management of their health. The greatest and statistically significant improvement is in feeling the they would be able to get the right help when they need it…” (Darnton et al., 2018a: 9)*  *“…involvement in the programme has also created a shift from being passive recipients of the medical care they receive to becoming more attuned to their own needs and better able to articulate them.” (Fullwood, 2018: 49)*  24. The patient is activated to manage their health (C) so is motivated to seek information about how to do this (M); consequently, they may make appointments to see their GP (O).  *“Patients were less anxious but their care was more costly and contact with primary care was not reduced.” (Grant et al., 2000: 423)*  *“…we can presuppose that attendance at a GP surgery may increase for a period of time following Navigation as patients engage more fully in managing* *their own conditions and identify solutions to their needs.” (Farenden et al., 2015: 36)*  *“…a statistically significant increase in the number of appointments completed in the three months after enrolment with PEP was observed. However, at 9 and 12 months after enrolment the number of appointments was significantly reduced.” (EMBED Health Consortium, 2016: 60)*  *“…other individuals stated that their GP use had not changed and for some their usage had increased due to having a greater awareness of their own health needs after engaging with the social prescribing service.” (Woodall et al., 2018: 7)*  “*Six months prior to signposting, there were a total of 41 GP visits among the patient cohort, at an average rate of 6.8 per month. Following signposting, the total number of appointments falls to 25. Note, however, that this number does not include a full 6 months “post intervention” period for all patients and the rate of visits per month is actually higher in the post intervention period (12.5 GP visits per month).” (RSM, 2017: 6)* |
| --- |
